# Controlling corporate influence in health policy making? An assessment of the implementation of article 5.3 of the World Health Organization framework convention on tobacco control

**DOI:** 10.1186/s12992-017-0234-8

**Published:** 2017-03-08

**Authors:** Gary Jonas Fooks, Julia Smith, Kelley Lee, Chris Holden

**Affiliations:** 1 School of Languages and Social Sciences, Aston University, Birmingham, UK; 2 Faculty of Health Sciences, Simon Fraser University, Vancouver, Canada; 3 Department of Social Policy and Social Work, University of York, York, UK

**Keywords:** Global health governance, World Health Organization Framework Convention of Tobacco Control, Corporate political influence, Treaty implementation, Article 5.3, Tobacco industry

## Abstract

**Background:**

The World Health Organization (WHO) Framework Convention on Tobacco Control (FCTC) stands to significantly reduce tobacco-related mortality by accelerating the introduction of evidence-based tobacco control measures. However, the extent to which States Parties have implemented the Convention varies considerably. Article 5.3 of the FCTC, is intended to insulate policy-making from the tobacco industry’s political influence, and aims to address barriers to strong implementation of the Convention associated with tobacco industry political activity. This paper quantitatively assesses implementation of Article 5.3’s Guidelines for Implementation, evaluates the strength of Parties’ efforts to implement specific recommendations, and explores how different approaches to implementation expose the policy process to continuing industry influence.

**Methods:**

We cross-referenced a broad range of documentary data (including FCTC Party reports and World Bank data on the governance of conflicts of interest in public administration) against Article 5.3 implementation guidelines (*n* = 24) for 155 Parties, and performed an in-depth thematic analysis to examine the strength of implementation for specific recommendations.

**Results:**

Across all Parties, 16% of guideline recommendations reviewed have been implemented. Eighty-three percent of Parties that have taken some action under Article 5.3 have introduced less than a third of the guidelines. Most compliance with the guidelines is achieved through pre-existing policy instruments introduced independently of the FCTC, which rarely cover all relevant policy actors and fall short of the guideline recommendations. Measures introduced in response to the FCTC are typically restricted to health ministries and not explicit about third parties acting on behalf of the industry. Parties systematically overlook recommendations that facilitate industry monitoring.

**Conclusion:**

Highly selective and incomplete implementation of specific guideline recommendations facilitates extensive ongoing opportunities for industry policy influence. Stronger commitment to implementation is required to ensure consistently strong compliance with the FCTC internationally.

**Electronic supplementary material:**

The online version of this article (doi:10.1186/s12992-017-0234-8) contains supplementary material, which is available to authorized users.

## Background

The World Health Organization (WHO) Framework Convention on Tobacco Control (FCTC) [1] stands to significantly reduce tobacco-related morbidity and mortality by accelerating the adoption of a comprehensive range of evidence-based policy instruments by signatory countries [2–7]. However, the extent to which States Parties to the Convention (Parties) have implemented specific commitments varies considerably. In 2014, WHO reported that 61% of reporting Parties had introduced complete smoking bans, and only half required pictorial warnings on tobacco products [8]. Civil society organizations (CSO) have consistently identified tobacco industry political influence as the key cause of weak FCTC implementation [9–14]. This is strongly supported by document based studies of tobacco companies’ political activities which illustrate their success over several decades in weakening, delaying, and preventing the introduction of public health measures [15–18]. News reports and academic studies of contemporary industry efforts to prevent implementation of the FCTC indicate that these risks are continuing [10, 13, 14, 19, 20]. In recognition of this, Article 5.3 of the FCTC – a highly innovative provision and the first of its kind in an international treaty - aims to address barriers to strong implementation of the Convention associated with tobacco industry political activity [7, 8, 21, 22]. The Article requires Parties to insulate the development and implementation of public health policies from tobacco industry influence [23]. Guidelines for Implementation of Article 5.3, published in 2008 [24], contain a number of recommendations (8 general and 34 specific) specifying how Parties should meet their obligations under the Article, which primarily focus on enhancing governments’ capacity to monitor the industry’s political activities and curb its insider political strategies (see Additional file 1: Table S1) [24]. In 2015, WHO reported over two-thirds of reporting Parties had taken steps to prevent the tobacco industry from interfering with tobacco-control policies [8]. Whilst this data suggests a significant increase on the 2012 reporting cycle [8], it only outlines whether Parties have taken some action under Article 5.3, and thus provides no indication of the strength of Parties’ efforts [21, 22], in terms of the number of recommendations acted upon (selective implementation), or the extent to which discrete recommendations are acted upon (incomplete implementation).

By combining data from reporting Parties with a wide range of other documentary data, this paper quantitatively assesses selective implementation of Article 5.3 guidelines, evaluates the strength of Parties’ efforts to implement specific recommendations, and explores how different approaches to implementation expose the policy process to continuing industry influence. Beyond this specific evaluation of Article 5.3 implementation, the wider purpose of the analysis is twofold: to provide a more detailed understanding of the continuing risks to health policy from industry political activity and inform efforts in other policy sub-systems - such as climate change, alcohol, and food policy - to minimise corporate political influence.

## Conceptual approach

Our analysis is based on the premise that selective and incomplete implementation of specific guideline recommendations provides residual opportunities for the industry to engage in political activity. By residual opportunities we refer to the unregulated administrative space created by selective and incomplete implementation, which either leaves existing political strategies unaffected or permits tobacco companies to adapt their strategies to take advantage of gaps in implementation (hereafter opportunities for policy influence unless stated otherwise). This assumption reflects a finding common to both the political science and public health literatures: corporations select political strategies with reference to the institutional and policy contexts in which they operate [25–31]. We seek to map the potential for opportunities for policy influence against a conceptual framework developed from studies on venue shopping [32–36], and corporate political activity based primarily on analyses of internal tobacco industry documents [16, 17].

Venue-shopping describes the practice of seeking out decision-making venues where social actors can best press their case for specific policy preferences [32, 33]. Internal document studies suggest that tobacco companies seek out alternative venues to supersede or circumvent health ministries (typically the lead department on FCTC implementation and a “veto point” for tobacco industry political activity [32, 33, 37–39]) with a view to exploiting different priorities of decision-makers in different fora [40, 41]. We adapt the well-established distinction in the literature, between horizontal and vertical venue-shopping, to differentiate between two types of opportunities for policy influence. *Vertical* opportunities arise along various levels of the policy-making process (e.g. government departments, legislature), while *horizontal* opportunities arise across sub-units within a given level of government (e.g. across different government departments) [32, 42, 43].

Studies of industry political activity highlight five other *tactically*-based opportunities that tobacco companies can use to acclimate to altered politico-institutional conditions, which derive from the heterogeneity and plasticity of tobacco industry political activity [16, 17]. The first are *functional-*opportunities which originate from tobacco companies’ capacity to trial qualitatively different techniques to optimise policy influence. These can work as alternatives to other techniques, in conjunction with them (such as voluntary marketing codes or youth smoking initiatives, which can be used to reinforce industry lobbying), or as direct substitutes (such as corporate social responsibility initiatives to gain informal access to difficult-to-reach policy élites) [17, 27–29, 44–46]. The second relate to *agent-based* opportunities, which derive from tobacco companies’ practice of using different actors either to optimise the credibility-leveraging effects of third parties, or because they are excluded from specific policy-making venues [17]. The third concerns tobacco companies’ capacity to politically innovate and adapt pre-existing techniques by changing their policy focus. In practice, these *focus-shifting* opportunities are primarily reflected in tobacco companies’ efforts to exploit government concerns over the illicit tobacco trade. This involves the use of strategic partnerships - historically used in the context of youth smoking - to facilitate access to policy élites and embed themselves in policy-making networks relevant to tobacco taxation [47–50]. Fourth, studies on tobacco companies’ efforts to shape trade and investment agreements [51–53], and embed cost-benefit analysis and risk assessment into policy decision-making [46, 54], demonstrate tobacco companies’ involvement in *venue-creation.* This involves the establishment of new forms, or levels of political governance (policy-making), which are not covered in the guidelines. Finally, informal methods of government implementation through uncodified, working norms may facilitate *temporal* opportunities for policy influence by allowing industry actors to take advantage of changes in political administrations or personnel over time.

For the sake of concision, our analysis of residual opportunity structures is illustrative, rather than exhaustive. Opportunities for policy influence do not represent exclusive categories. Tobacco companies, for example, may trial different techniques in different policy-making venues by deploying third parties. In such cases, opportunities for policy-influence are functional, venue *and* agent-based. Equally, tobacco manufacturers typically partner revenue-raising ministries with respect to the illicit trade, an opportunity which is both focus-shifting and venue-based. Nevertheless, we present the various residual opportunities for policy influence that arise from selective or incomplete implementation as discrete categories. Further, in our quantitative analysis of selective implementation, we exemplify the different forms of opportunity, rather than provide a comprehensive account of the full range that theoretically persists. Likewise, our thematic analysis of the strength of implementation of specific recommendations focuses on proximate venue and agent-based opportunities that arise in relation to the specific political technique targeted by the recommendation in question. Finally, we leave discussion of continuing opportunities facilitated by gaps in the guidelines to the conclusion.

## Methods

Article 21.1 of the FCTC requires Parties to submit periodic reports to the Conference of the Parties through the Convention Secretariat on implementation [55]. WHO’s reporting template provides Parties with an option to designate (yes or no) whether they have measures in place “protecting public health policies with respect to tobacco control from commercial and other vested interests of the tobacco industry” [56]. Parties can also provide details on measures taken in accordance with Article 5.3, and any progress on implementation since the last reporting period [56]. In addition, parties are invited to submit answers to additional questions on implementation [57].

At the time of analysis, there were 179 Parties to the FCTC. We downloaded reports for 167 Parties from the WHO database between October 2014 and February 2015 (reports from 11 State Parties were not accessible, 1 related to the European Union and was excluded for consistency of comparisons). Reports not in English were translated using Google Translate. Parties whose languages could not be translated (*n* = 12) were excluded. Of the remaining 155 Parties (n_Africa_ = 36; n_Americas_ = 28; n_Asia_ = 49; n_Europe_ = 42), 104 (67%) answered yes to whether they had undertaken measures in accordance with Article 5.3 in their latest report, 92 included additional narrative detail, and 19 submitted answers to additional questions.

## Data collation

The analysis drew upon a range of sources: Parties’ commentaries in their periodic reports on measures taken in accordance with Article 5.3; tobacco control legislation, regulations, codes of practices, decrees made available in the WHO party reporting database, and an online collection of legal and administrative documents maintained by the International Legal Consortium and Campaign for Tobacco-Free Kids (http://www.tobaccocontrollaws.org/); data on the governance of political financing collated by the International Institute for Democracy and Electoral Assistance [58]; World Bank material on the governance of conflicts of interest in public administration [59] and the United Nations Economic and Social Council on implementation of the International Code of Conduct for Public Officials [60]; existing studies on lobbying regulation [61–66]; reports by CSOs on industry interference in policy-making [67, 68]; and emails to contact officers named in Party reports.

Emails were sent to all 104 Parties that had reported taking some action under Article 5.3. Emails contained 7 general questions seeking further information on Article 5.3 implementation covering, for example, the specific methods used to put recommendations into effect. In 27 of these emails, supplementary questions sought clarification on specific points raised in the narrative sections of Parties’ reports. Emails were translated into Parties’ official languages using Google Translate with the English version also included below the translated copy. Nine responses were received from the first batch of emails. Follow-up emails were sent to all Parties that had not responded, repeating the original questions and asking for any further documentation. These second emails generated 6 further responses.

## Data analysis

We evaluated the strength of Article 5.3 implementation with reference to two indicators which provide a basis for exploring residual opportunities for policy influence: a) the number of specific recommendations acted upon by each Party (which provides a basic method for exploring functional opportunities for policy influence); and b) the strength of specific measures undertaken compared to specific guideline recommendations (which provides a basis for exploring venue, functional, agent, focus-shifting, and temporal based opportunities). This evaluation involved two types of in-depth analysis: a cross-referencing exercise (undertaken by JS and GJF) between measures undertaken and specific guideline recommendations; and an interpretative analysis (undertaken by JS and GJF) of the most commonly implemented recommendations aimed at identifying conceptual themes relevant to the strength of specific measures carried out. For both exercises, we defined *implementation* in terms of formally approved (but not necessarily in effect) policy instruments (codes of practices applicable to public and elected officials, administrative measures, and primary and secondary legislation) as of January 2015; and uncodified *working norms* (hereafter collectively described as measures unless otherwise stated). We define w*orking norms* to be in place when a party claims to implement a recommendation (or set of recommendations) methodically, or successfully takes *ad hoc* action, consistent with a recommendation in the absence of a formal policy instrument. Where relevant we distinguish between the two in the interpretative analysis.

For the cross-referencing exercise JS and GJF independently read the collated data for measures which broadly corresponded to specific guideline recommendations. Many recommendations combine several discrete measures. Parties were given a score of one for each recommendation partially or fully acted on and zero where measures were not implemented (with the exception of prohibitions on tobacco sponsorship limited to promoting or inducing the use of tobacco products which were coded as zero). We assigned a score of zero where Party reports appear to incorrectly claim that a specific recommendation has been implemented in a law or regulation and where implementing measures recommend, rather than require, officials to act in accordance with a recommendation. Ten recommendations were excluded from the analysis (leaving a total of 24 recommendations) to take account of our interest in efforts to reduce the impacts of industry political activity and provide a reliable framework for comparison between Parties: four of these recommendations (4.1, 4.2, 5.1 and 5.5) are broadly defined and overlap with others; one (5.4) centres on enforcement; three (8.1-8.3) are concerned with state-owned tobacco enterprises (the majority of which have now been privatized [69]); and two (7.1 and 7.3) relate to state subsidies. We also disregarded sections of discrete recommendations which do not directly relate to industry political activity (see aspects of recommendations 1.1 and 5.2 in Additional file 1: Table S1) [24]. The data were entered into an Excel spreadsheet. Scoring was discussed at regular intervals to ensure consistency and reach consensus on divergent views.

The interpretative analysis applied the techniques of thematic analysis outlined by Guest, et al*.* [70]: conceptual coding, theme development, systematic conceptual comparison, and conceptual explanatory conclusions [70]. GJF read and coded the data for relevant themes. Coding was inductive and emergent and informed by the terms of specific recommendations (particularly composite recommendations which urge Parties to implement a number of discrete measures), the literature on tobacco industry political activity [16, 17] and venue-shopping [32–35]. Reports were downloaded into QSR NVivo 10 and specified measures were micro-coded for conceptual ideas relevant to the capacity of the measure to limit industry interference in health policy-making. Coding was discussed at regular intervals with JS to ensure consistency and reach consensus on divergent views. When coding was completed, the codes were re-examined for conceptual coherence and clarity and examined for relationships among them.

The study has several weaknesses which may understate the strength of implementation. First, Party reports represent a poor source of data. WHO’s reporting template does not provide in depth guidance on how guideline recommendations should be reported on. Some Parties include irrelevant information, suggesting poor understanding of Article 5.3. Both factors may result in Parties failing to report measures consistent with Article 5.3. Second, the relatively low response rate to our email queries (likely to have been exacerbated by our reliance on Google Translate), combined with our focus on documentary data, is likely to underrepresent the extent of implementation achieved through uncodified working norms. Third, not all World Bank data on the governance of conflicts of interest in public administration is publicly available. We sought to take account of this by conducting internet searches for legal instruments (including codes of conduct) for Parties not in the World Bank dataset. Nonetheless, the findings are likely to underrepresent the extent to which Parties have codified recommendations 4.3-4.10. Fourth, the study did not review existing freedom of information laws. Despite research indicating that they represent poor tools for accessing information on lobbying, either because contacts are not documented (and, therefore, the data does not exist) or because of exemptions concerning commercially sensitive information [66], in some countries these may provide CSOs with access to information concerning industry-government interactions in accordance with recommendation 2.2. Finally, we did not systematically examine implementation at the regional or local levels, which potentially provide important opportunities for policy influence in federal systems of governance. The study was approved by Aston University’s Languages and Social Sciences ethics committee.

## Results

### Overview of compliance and implementation

#### Methods of implementation

**Fig. 1:**
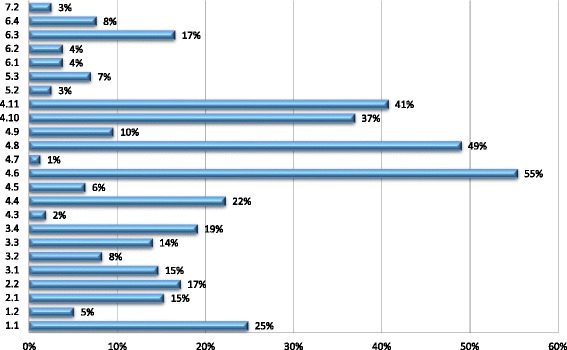
Frequency of Implementation by Recommendation (for all parties in sample)

**Fig. 2:**
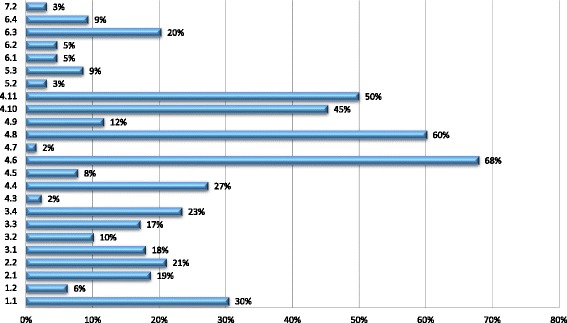
Percentage Implementation by Recommendation (for parties that have taken some steps in accordance with Article 5.3)

Implementation is primarily *passive*. It is achieved through existing policy instruments introduced independently of the FCTC which govern conflicts of interest in public administration, political financing, and transparency in lobbying and which reflect recommendations 4.4, 4.6, 4.8, 4.10 and 4.11 (see Additional file 1: Tables S2 and S3, Figs. 1 and 2). *Active* implementation, which involves purposeful action to implement the Article, is typically codified in policy instruments that reflect specific guideline recommendations and focus on managing the behaviour of policy actors. Some Parties have also created general legal duties which aim to minimise industry involvement in health policy-making. Where this occurs, Parties commonly reproduce the wording of Article 5.3 alongside other, more targeted, measures (Djibouti [71], Mongolia [72], the Philippines [73]). However, Ukraine’s main tobacco law [74] potentially creates a power to challenge demonstrable industry influence (as opposed to industry political activity) by giving health protection priority over “financial, tax, and corporate interests of economic subjects” [74], whilst Panama has taken an intermediate approach by empowering the Ministry of Health to establish a commission with responsibility for recommending, instituting, and monitoring compliance with the general guideline recommendations [75]. Other Parties have also introduced broad, industry-focused duties. Gabon’s recent tobacco control law [76], for example, requires the industry and related third parties to conduct themselves in a responsible and transparent fashion. Finally, some Parties forgo codification by either introducing working norms that reflect specific recommendations (see Additional file 1: Tables S5, S8, S9) or by making general, uncodified commitments to act in accordance with Article 5.3. In relation to the latter, Colombia, has adopted the protection of public health policies from industry interests in its public health plan [77], whereas the Netherlands claims to “act within the spirit of the guidelines” [78] on issues such as industry government interactions and involvement of the industry in tobacco control policy [78].

The basic design features of some of the above approaches to implementation can limit their impact on industry opportunities for policy influence. Active implementation, for example, is commonly restricted to governing the behaviour of health officials and tobacco companies (see, for example, Additional file 1: Tables S5 and S9), which sustains horizontal and vertical venue-based opportunities for policy influence. Equally, implementation that focuses exclusively on operationalising specific recommendations fails to take account of the industry’s capacity to innovate politically (see Conclusions). General and industry-focused duties may offset this effect; future-proofing policy-making against innovation by giving policymakers and civil society actors greater scope to contest new forms of policy influence. However, uncodified general commitments are more open to being rescinded by new political administrations - creating greater temporal opportunities for policy influence - and are likely to be more difficult to enforce. This interpretation finds some support from a recent legal summons initiated by the Youth Smoking Prevention Foundation in the Netherlands, which claims that Article 5.3 is routinely ignored [79, 80].

#### Strength of implementation

Party reports underestimate the number of Parties that have taken some action to implement Article 5.3. One hundred and four (67%) [n_Africa_ = 23(64%); n_Americas_ = 17(61%); n_Asia_ = 36(73%); n_Europe_ = 28(67%)] Parties in our sample report to have taken steps to implement Article 5.3. However, misreporting is extensive, reflecting poor understanding of Article 5.3 and limited awareness of existing policy instruments consistent with the guideline recommendations. Twenty-eight Parties include some information unrelated to Article 5.3 in the relevant narrative section of their reports, whilst 31 Parties report no action despite having measures in place relating to conflicts of interest in public administration and financing of political parties which reflect one or more guideline recommendations. One hundred and twenty-eight (83%) [n_Africa_ = 28(78%); n_Americas_ = 21(75%); n_Asia_ = 41(82%); n_Europe_ = 38(90%)] have measures in place which are consistent with Article 5.3 and its guidelines once misreporting is taken into account.

**Fig. 3:**
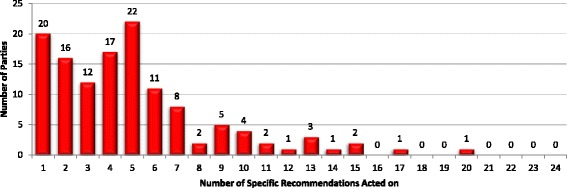
Number of Specific Recommendations Implemented by Party (parties that have taken some steps in accordance with Article 5.3)

Despite the fact that more Parties have taken some action under Article 5.3 than suggested by WHO reports, effective implementation of the Article is generally weak. Additional file 1: Tables S2 and S3 outline the total number of recommendations acted on by all Parties in the sample (Additional file 1: Table S2) and by those that have taken some action in accordance with Article 5.3 (Additional file 1: Table S3). The data indicate that only 16% of the recommendations reviewed have been implemented (Additional file 1: Table S2). The percentage only rises to 20% when Parties that have not taken steps in accordance with Article 5.3 are excluded (Additional file 1: Table S3). Simple frequency data indicate that *de minimis* implementation, where Parties implement a small number of recommendations, is relatively widespread and implementation generally is highly selective. 38% of Parties that have taken some steps in accordance with Article 5.3 have implemented ≤3 specific recommendations, whilst just under 83% (n_parties_ = 106) have introduced less than a third (n < 8) (Fig. 3). In fact, only 8 Parties, just over 6%, have introduced more than half (n > 12) (Fig. 3).

Parties that fail to implement specific recommendations create an unregulated environment for the specific forms of industry political activity those recommendations seek to address. *De minimis* implementation potentially has more fundamental effects. First, it creates a permissive environment for functional opportunities for policy influence where key techniques of political activity escape regulation altogether. For example, implementation of recommendation 2.2 can partially offset the effect of failing to restrict government-industry interactions in accordance with recommendation 2.1 (Additional file 1: Tables S1 and S3, Fig. 2), by ensuring that interactions that do occur are transparent. However, in practice recommendations 2.1 and 2.2 are commonly implemented in tandem (Africa, 25%; Americas, 67%; Asia, 62%; Europe, 38%), with the effect that industry-government interactions are completely unregulated in most cases (Africa, 89%; Americas, 59%; Asia, 76%; Europe, 69%). Second, selective and *de minimis* implementation facilitates agent-based opportunities for policy influence by permitting tobacco companies to make use of third parties without effective scrutiny. This is a consequence of low rates of implementation of recommendations 5.2 and 5.3, which urge Parties to introduce rules of disclosure and reporting requirements for organizations affiliated to the industry or acting on its behalf (see Additional file 1: Table S1, S2 and S3, Figs. 1 and 2).

#### Awareness raising of industry political activity (recommendations 1.1, 1.2, and 6.1)

*1.1 Parties should…inform and educate all branches of government and the public about the….need to protect public health policies for tobacco control from commercial and other vested interests of the tobacco industry and the strategies and tactics used by the tobacco industry to interfere with the setting and implementation of public health policies with respect to tobacco control.*

*1.2 Parties should, in addition, raise awareness about the tobacco industry’s practice of using individuals, front groups and affiliated organizations to act, openly or covertly, on their behalf or to take action to further the interests of the tobacco industry.*

*6.1 Parties should ensure that all branches of government and the public are informed and made aware of the true purpose and scope of activities described as socially responsible performed by the tobacco industry.*

Recommendations 1.1, 1.2 and 6.1 urge Parties to undertake awareness raising activities to increase pan-governmental and public surveillance of industry political activity and ensure a whole-of-government approach to minimising the industry’s opportunities for policy influence by changing officials’ behaviour towards their political activity. Recommendation 1.1 represents a general provision aimed at encouraging Parties to educate government departments and publics about the nature of industry political activity. Recommendations 1.2 and 6.1 specifically encourage Parties to extend awareness raising to tobacco manufacturers’ use of third parties and corporate social responsibility (CSR) initiatives to influence policy. In practice, variations in how awareness raising activities are organised (relating to their method of delivery, target audience and content), and the institutional contexts in which they take effect, are likely to have a significant bearing on their effectiveness.

Parties have trialled six methods of awareness raising (Additional file 1: Table S4): pan-governmental administrative circulars (circulars); meetings, workshops, presentations, and consultations (meetings); training based measures (training), in which awareness raising is embedded in the training of civil servants and legal advisers of government ministries; intra-governmental advocacy by health ministries (or tobacco control agencies) aimed at providing intelligence to other parts of government targeted by the industry in the context of specific policy conflicts (intra-governmental advocacy); ongoing campaigns by national, regional, and local health officials aimed at highlighting the policy value of protecting health policy from industry interference (campaigning); and mass media campaigns which use local television advertisements, newspaper articles, radio call in shows, and websites to raise awareness of industry interference among the general public (public awareness raising). These activities address four broad audiences: officials from central and local/regional government and government agencies; elected representatives; non-state actors, specifically civil society organizations (CSOs), journalists and community leaders; and the general public (Additional file 1: Table S4).

It is difficult to draw firm conclusions about the impact of different methods of awareness raising as few parties monitor their effects on officials’ behaviour (but see [81]). Awareness raising activities through the mass media may help build political support for stronger restrictions on, and systems for monitoring industry political activity. Equally, the timely and targeted nature of intra-governmental advocacy may help to develop working norms among policy actors that are consistent with the aims of Article 5.3. Brazil reports that intra-governmental advocacy by Comissão Nacional para Implementação da Convenção - Quadro para Controle do Tabaco (CONICQ) (a national commission for FCTC implementation comprising representatives from across the federal government) in the context of specific policy conflicts has been effective in countering industry lobbying of non-health departments [82]. However, research on the effects of education on professionals’ behaviour in the workplace raises doubts about the effectiveness of approaches, which are not ongoing, learner-centred, and interactive [83, 84] such as one-off meetings and the passive dissemination of information through pan-governmental circulars. The effectiveness of meetings will also be dependent on whether participants have sufficient institutional capital to influence policy-making norms (as a result of seniority or professional expertise), and are drawn from across public administration. Officials from departments whose institutional priorities are governed by the current revenue generated by the tobacco industry and tobacco consumption (such as finance ministries and customs and excise departments, or departments responsible for brokering international trade and investment agreements), elected representatives, and legal advisers to government departments, all play important roles in shaping health policy and, therefore, frustrating tactical adaptation by the industry [41]. Reports typically exclude this information.

The quality and relevance of information provided in awareness raising activities is also relevant to their effectiveness in controlling tactical and functional adaptation. Much industry political activity is opaque, which poses acute challenges to policymakers. The industry’s reliance on third parties, for example, makes it difficult for public officials to identify industry interests and respond accordingly [16, 17, 85, 86]. Likewise, industry CSR programmes and liaison with governments over the illicit trade in tobacco often appear unconnected from policy influence [27–29, 44, 45, 87], which can obscure the political risks of engagement [48, 88, 89]. Finally, the poor evidential quality of tobacco manufacturers’ submissions to public consultations is buried beneath non-scientific and misleading epistemological techniques which create a parallel ‘scientific’ discourse that is difficult to unpick without extensive forensic examination [90, 91]. Given the growing use of mandatory regulatory impact assessments [46, 92] which place administrative obligations on policymakers to give serious consideration to industry representations concerning the relative costs and benefits of health policies, officials are increasingly required to engage with and effectively disaggregate these techniques if health policies are to be shielded from an important form of functional adaptation not explicitly covered by the guidelines. Effective awareness raising must therefore outline the range of techniques and actors used in industry political activity, specify the identity of actors with links to tobacco manufacturers, and provide detailed accounts of the techniques manufacturers’ use to provide a scientific veneer to poor quality information: issues, which are likely to vary between states and policies [17]. There is little evidence to suggest that these issues are systematically addressed in practice.

Finally, institutional contexts influence the effectiveness of awareness raising by setting limits on the practical value of policymakers’ increased mindfulness of industry political activity. Thus, the extent to which awareness raising can help policymakers identify third parties acting on behalf of the industry is highly dependent on effective procedures for monitoring the industry’s political actions or requiring tobacco manufacturers to make transparent their political activities, which few Parties have acted on. Equally, awareness raising will have little practical effect in the absence of administrative levers to put enhanced awareness of industry political activity into effect – such as administrative codes aimed at limiting industry-government interactions, industry involvement in policy-making, or conflicts of interest.

### Government and industry interactions (recommendations 2.1 and 2.2)

#### Restricting access to policymakers

*2.1 Parties should interact with the tobacco industry only when and to the extent strictly necessary to enable them to effectively regulate the tobacco industry and tobacco products.*

By encouraging Parties to restrict industry actors’ access to public officials, recommendation 2.1 aims to shield public and elected officials from poor quality and misleading industry information and reduce opportunities for industry actors to cultivate more co-operative, higher trust relationships with policymakers [28]. The effectiveness of restrictions depends on the extent to which they take account of the methods used by the industry to lobby officials which rely heavily on third parties [93, 94] and focus on departments concerned with revenue generation, intellectual property considerations, and trade and investment relations [16, 17, 40, 48, 51, 52, 89, 95, 96] and heads of state [96], as well as health ministries.

Restricting access to non-health departments is particularly important given that they can have a stronger voice in government [40, 48] and fewer reservations over the value of industry information [29, 40, 41, 48, 51, 95, 97]. Norway’s Ministry of Finance, for example, liaises with the industry to obtain information on illicit tobacco sales and smuggling, which it uses to set tax levels despite doubts over the accuracy of such information [98–102]. Differing perceptions of the reliability of industry information among business and finance related departments may reflect their relative remoteness from transnational networks, such as the Framework Convention Alliance, which seek to highlight the poor quality of industry information. Moreover, routine interactions between industry actors and officials from these departments is also likely to facilitate more trusting, co-operative relationships which further strengthens perceptions of the credibility of industry information [103]. In practice, restrictions rarely seem to be extended to interactions with third parties or with officials beyond health ministries (Additional file 1: Table S5), implying that (horizontal and vertical) venue and agent-based opportunities for policy influence persist even in jurisdictions with constraints on government-industry exchanges.

A number of Parties have also developed detailed administrative rules, codifying how industry-government interactions should take effect (Additional file 1: Table S6). In addition to restricting industry actors’ capacity to shape the agenda of meetings with officials [29] the existence of such rules limits the potential for informal, unregulated, exchanges (in fact, Philippines’ rules advocate that all “non-mediated exchanges” should be avoided [104]) and, by creating obstacles to industry access, provides a deterrent to industry lobbying. Their practical impact, however, is moderated by their limited institutional reach and the fact that they rarely apply explicitly to third parties (Additional file 1: Table S6).

#### Transparency of industry-government interactions

*2.2 Where interactions with the tobacco industry are necessary, Parties should ensure that such interactions are conducted transparently. Whenever possible, interactions should be conducted in public, for example, through public hearings, public notice of interactions, disclosure of records of such interactions to the public.*

Although many measures aimed at making government-industry interactions transparent apply across government, recommendation 2.2 is weakly implemented. Few measures make high-quality, easily accessible information available for public scrutiny and, consequently, are unlikely to significantly affect existing opportunities for policy influence.

By giving politically influential actors direct, low-cost access to high quality information, transparency protocols that give observer status to CSOs potentially represent the most effective method for reducing industry influence (Additional file 1: Table S7). However, this approach is rare. Most Parties seek to implement recommendation 2.2 by placing details of industry-government communications in the public domain. Variations in this approach produce wide disparities in the accessibility and quality of information available for public scrutiny. Few measures give the public unrestricted access to minutes of interactions that reveal the substance of discussions (Additional file 1: Table S7). Most Parties simply publish basic details of meetings (such as dates, those in attendance, and broad topics discussed). This is likely to impede effective monitoring by requiring CSOs to submit requests for minutes under FOI laws, which not only increases the costs of monitoring, but also commonly contain exemptions which preserve the confidentiality of industry-government interactions [105]. Even where confidentiality exemptions are subject to public interest tests FOI may still represent a weak instrument for enhancing transparency. In the UK, for example [106], where confidentiality exemptions in FOI legislation are subject to a public interest test, ministers are not required to declare meetings with lobbyists if they take place in their private time or constituency roles [107], and the Regulatory Policy Committee, a strategically important body in assessing the costs of regulation [108], is not covered by the Freedom of the Information Act (2000). Just as importantly, transparency protocols that focus on meetings exclude correspondence and telephone conversations. Further, in some cases details of meetings are only available on request (Additional file 1: Table S7). Finally, many Parties’ protocols, neither apply to interactions across government nor to third parties acting on behalf of the industry (Additional file 1: Table S7): gaps which are likely to assume greater practical significance if restricted access to policymakers within health departments cause industry actors to shift the focus of lobbying to other departments.

A number of Parties (Austria, Cyprus, Estonia, France, Lithuania, the Netherlands, and Slovenia) require publication (under certain circumstances) of the names of persons consulted in respect of draft legislation. However, comprehensive legislative footprints are rare (see Additional file 1: Table S7 with respect to Latvia and Poland) and only apply to draft laws introduced before legislatures rather than to policy-making generally [109].

### Partnerships, self-regulation, and legislative drafting (recommendations 3.1-3.4)

*3.1 Parties should not accept, support or endorse partnerships and non-binding or non-enforceable agreements as well as any voluntary arrangement with the tobacco industry or any entity or person working to further its interests.*

*3.2 Parties should not accept, support or endorse the tobacco industry organizing, promoting, participating in, or performing, youth public education or any initiatives that are directly or indirectly related to tobacco control.*

*3.3 Parties should not accept, support or endorse any voluntary code of conduct or instrument drafted by the tobacco industry that is offered as a substitute for legally enforceable tobacco control measures.*

*3.4 Parties should not accept support or endorse any offer for assistance or proposed tobacco control legislation or policy drafted by or in collaboration with the tobacco industry.*

Recommendations 3.1-3.3 urge Parties to formally eschew industry efforts to pre-empt statutory regulation though partnerships and other forms of strategic voluntary or self-regulation which fill regulatory space, reduce political support for strong marketing and tax policies, and foster closer relationships between industry and policy actors. Few Parties appear to have formal protocols prohibiting these voluntary measures (Additional file 1: Table S8). Parties’ responses to the additional questions (which ask whether Parties have entered into partnerships, non-binding, non-enforceable, or voluntary arrangements with the industry and whether they are aware of any youth, public education, or other initiatives related to tobacco control) are consistent with a greater incidence of informal working norms (Additional file 1: Table S8). However, it is not clear whether this is a function of self-selection bias (Parties with stronger records on Article 5.3 being more likely to submit responses to the questions) or whether Parties’ responses take into account partnerships and voluntary arrangements relating to the illicit trade in tobacco. If not, the potential for focus-shifting opportunities is likely to be significant [110].

By urging Parties to refuse offers for assistance in respect of proposed tobacco control legislation or policy drafted by (or in collaboration with) the tobacco industry, recommendation 3.4 seeks to shield policymakers from poor quality information which ostensibly appears to reduce the costs associated with developing tobacco control policies [17]. Stronger, formal methods (*n* = 12) are put into effect through primary and secondary legislation, and administrative measures (Additional file 1: Table S9). Informal methods – which are susceptible to temporal opportunities for policy influence – include working norms (*n* = 11) and ad hoc actions (*n* = 7). The degree of specification of formal protocols varies considerably, which may affect their value to policy actors seeking to rely on them to ensure offers of assistance are rejected (see Nepal’s Tobacco Product Control and Regulatory Directive [111] for an example of a highly specified approach). Only 4 Parties explicitly extend their protocols to third parties (Additional file 1: Table S9), while fewer still (*n* = 2) have drafted formal protocols that explicitly extend to non-health ministries (Additional file 1: Table S9); creating both agent and venue-based risks of policy influence [41].

### Managing and making transparent individual conflicts of interest (recommendations 4.4, 4.6, 4.8, 4.9, 4.10)

#### Engagement in occupational activity within the tobacco industry after leaving service

*4.4 Parties should develop clear policies that require public office holders who have or have had a role in setting and implementing public health policies with respect to tobacco control to inform their institutions about any intention to engage in an occupational activity within the tobacco industry, whether gainful or not, within a specified period of time after leaving service.*

Recommendation 4.4 seeks to reduce the impact of the “revolving door” phenomenon by requiring officials to disclose any intention to work within the industry once leaving public service. Offers of employment have the potential to affect current policy-making through the promise of future financial inducements [112], by enhancing insider knowledge of policy-making [113], and by increasing industry access to policymakers through personal ties [114]. In practice, some measures (n_Parties_ = 7) require policy actors to disclose future employment plans, but most focus on imposing post-employment restrictions: prohibiting policy actors from taking up certain forms of employment within a specified period of time (cooling-off provisions) (n_Parties_ = 27) or from lobbying (n_Parties_ = 3).

Most in-post disclosure obligations have basic drafting flaws which provide scope for industry tactical and venue adaption. Not all apply to senior policymakers (such as ministers (n_Parties_ = 6) or heads of state (n_Parties_ = 3)) who have the institutional power to act as veto players in health policy-making, and few apply across all levels of policy-making (n_Parties_ = 2). Although most measures that impose post-employment restrictions apply across public administration (91%, n_Parties_ = 27) they are subject to similar weaknesses, which limit the extent they can curtail employment as a means of policy influence. Few cooling-off provisions, for example, apply to senior policy actors (ministers (48%); heads of state (34%)). Fewer still (24%) apply across all levels of policy-making (heads of state, ministers, elected representatives, and civil servants). Selective application is compounded by: the absence of disclosure obligations for cooling-off provisions (which apply to just 24% of policy actors subject to such provisions); short cooling-off periods of 6 months for some policy actors (n_Policy Actors_ = 7); narrow restrictions on the types of employment (e.g. board membership) (n_Policy Actors_ = 11) and types of employers (e.g. those that have been “controlled” or “regulated” by policy actors) (n_Policy Actors_ = 25) that give rise to cooling-off periods. Just 5 Parties apply comprehensive cooling-off periods (more than 6 months, across all levels of policy-making and not unduly restricted to specific types of employment or employers) or in-post disclosure requirements which apply across all levels of policy-making.

#### Declaration and divestment of direct interests in the tobacco industry and excluding tobacco industry employees from health policy-making

*4.6 Parties should require government officials to declare and divest themselves of direct interests in the tobacco industry.*

*4.8 Parties should not allow any person employed by the tobacco industry or any entity working to further its interests to be a member of any government body, committee or advisory group that sets or implements tobacco control or public health policy.*

*4.9 Parties should not nominate any person employed by the tobacco industry or any entity working to further its interests to serve on delegations to meetings of the Conference of the Parties, its subsidiary bodies or any other bodies established pursuant to decisions of the Conference of the Parties.*

Recommendation 4.6 urges Parties to require public officials to declare and divest themselves of “direct interests in the tobacco industry”: a broadly defined term which, in principle, extends to firm and share ownership as well as various forms of secondary employment, such as board, management and advisory roles. The recommendation overlaps with, and reinforces, two further recommendations which are not restricted to public officials: recommendation 4.8, which seeks to prevent industry employees (and those working to further their interests) from sitting on government bodies that set or implement public health policy; and recommendation 4.9 which seeks to extend a similar protection to international policy-making by discouraging Parties from nominating industry actors from serving on delegations to meetings of the Conference of the Parties, its subsidiary bodies or other bodies established pursuant to its decisions. A key difference between recommendation 4.6 and recommendations 4.8 and 4.9 is that the latter cover non-public employees who may participate in policy-making on a less formal basis.

Implementation of recommendations 4.6 and 4.8 is primarily passive and codified, whereas recommendation 4.9 is typically implemented “actively” through working norms. We identified just 11 health-based policy instruments introduced in response to the FCTC relevant to recommendations 4.6 and 4.8 (which in most cases, n_Parties_ = 8, sit alongside measures that apply to policy-making generally) and 1 relevant to recommendation 4.9. This may, however, underestimate active implementation of recommendation 4.8 and 4.9, which, in contrast to recommendation 4.6, can be put into effect on an ad hoc basis (e.g. Ghana’s actions over participation in meetings of its Tobacco Control Inter-Agency Coordinating Committee [115]). This is implied by Parties’ answers to the additional questions, which indicate that observance of recommendation 4.8 and 4.9 through working norms and ad hoc actions may be relatively widespread.

Health-based policy instruments covering recommendation 4.6 and 4.8 tend to be institutionally selective; focusing on the membership of bodies established to govern tobacco control policy (e.g. [116–119]). This narrow focus creates an enabling environment for horizontal and vertical venue-based opportunities for policy influence. In 2010, for example, the tobacco industry’s participation in Latvia’s State Committee on Restriction of Smoking was prohibited by statute [120, 121]. At the same time, however, someone with links to Philip Morris is reported to work in the Department of Taxation Policy based in the Ministry of Finance [122]. Despite this, obligations relating to the objects of divestment in health-based instruments are broadly defined in most cases (Kenya’s Tobacco Control Act [2007], for example, prohibits members of the country’s Tobacco Control Board from being “directly or indirectly…affiliated to the tobacco industry or its subsidiaries”) and accompanied by disclosure requirements [116].

**Fig. 4:**
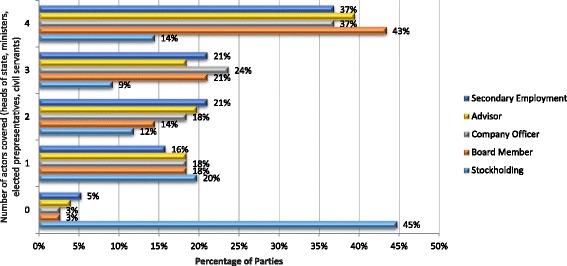
Policy Instruments restricting public officials’ external interests (by policy actors covered)

**Fig. 5:**
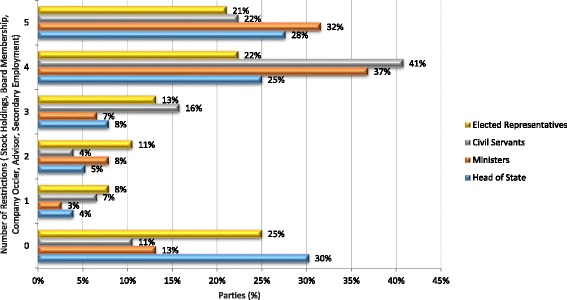
Policy Instruments restricting public officials’ external interests (by restrictions on interests imposed)

Policy instruments that apply across public administration, and which are consistent with recommendations 4.6, 4.8 and 4.9, work by imposing restrictions and prohibitions on policy actors’ external interests: specifically, stock holdings/firm ownership, board membership, employment as a company officer, acting as a firm advisor, and general secondary employment. These instruments sustain an accommodating environment for conflicts of interests for several reasons. First, they are usually restricted to public employees, and, therefore, do not cover industry actors who may contribute to health policy-making on a more informal basis. Second, because they develop in a piecemeal way, consolidated instruments that apply to all public and elected officials are relatively rare. As a result, the percentage of Parties with policy instruments that cover heads of state, ministers, elected representatives, and civil servants is relatively low (≥43% for all interests covered) (see Fig. 4). Third, the full range of external interests that may give rise to conflicts of interests are rarely covered. In some cases, for example, prohibitions only extend to professional activities, managing businesses, paid employment, or company stock holdings, or particular modes of stock holding. This is illustrated by Fig. 5 which shows that strong provisions extending to the full range of interests only apply in 32% of cases for ministers, 28% for heads of state, 22% for civil servants, and 21% for elected representatives. Finally, in some cases restrictions on stockholdings are subject to provisos and qualifications. Armenia’s law on the Civil Service (2001), for instance, requires civil servants to pass control of stocks in a commercial organization to entrusted management (which does not affect the officials’ right to receive income from the property) where his or her share in the company exceeds 10%.

#### Prohibition of gifts, services and benefits

*4.10 Parties should not allow any official or employee of government or of any semi/quasi-governmental body to accept payments, gifts or services, monetary or in-kind, from the tobacco industry.*

Recommendation 4.10 urges Parties to prohibit government officials and employees from accepting gifts, payments and services (hereafter gifts) of any kind from the tobacco industry. Measures that specifically target tobacco industry gifts are uncommon (n_Parties_ = 3) and, in practice, simply reproduce existing measures introduced independently of the FCTC that apply across public administration [73, 123]. Passive implementation is more common (n_Parties_ = 61), but measures are characterised by exemptions, provisos, and qualifications which provide ample scope for industry tactical adaption. Potential veto players, for example, are commonly excluded [heads of state, 44% (n_Parties_ = 27); ministers, 20% (n_Parties_ =13)]. Equally, some measures impose monetary thresholds on the value of gifts that can lawfully be accepted [heads of states (n_Parties_ =9); ministers (n_Parties_ = 11); civil servants (n_Parties_ =11)]. Limits are typically set quite low (e.g. US$8 in Croatia), but higher thresholds (e.g. Mongolia, monthly salary of the public official; Georgia, 5% of public officials’ annual income; Turkey, 10 times the salary of government ministers) are likely to have little impact on tobacco companies’ capacity to use personal gifts as a lawful means of influencing policymakers’ decision-making. Exemptions are also common [heads of states (n_Parties_ = 8); ministers (n_Parties_ = 11); civil servants (n_Parties_ = 12)], which in some cases can appear quite significant (in Palau, for example, wedding gifts, customary gifts and gifts exchanged between individuals on birthdays, holidays and other similar occasions are permitted provided the gifts exchanged are not substantially disproportionate in value).

### Managing Institutional Conflicts of Interest: Political Financing (Recommendations 4.11)

*4.11 Taking into account national law and constitutional principles, Parties should have effective measures to prohibit contributions from the tobacco industry or any entity working to further its interests to political parties, candidates or campaigns, or to require full disclosure of such contributions.*

Recommendation 4.11 covers political financing. It urges Parties to either prohibit businesses in the tobacco supply chain (or those working to further their interests) from financing political parties, candidates, or campaigns or require their full disclosure. Although uncommon (n_Parties_ = 3), measures that target tobacco industry political financing are comprehensive, banning contributions to both parties and candidates [124–127]. By contrast, measures that apply generally to political funding (n_Parties_ = 89) commonly fall short of comprehensive implementation. Although 50 Parties have measures in place that impose restrictions on political contributions, only 30 prohibit contributions to both parties and candidates, which provides tobacco companies with functional-based opportunities for influence by extending funding to candidates where party financing is prohibited and vice-versa. Further, bans typically work by either limiting political funding to natural persons (individuals) or prohibiting legal persons - such as companies - from financing parties and candidates. They do not, as such, cover donations from individual shareholders or other natural persons who may wish to promote the interests of businesses in the tobacco supply chain. Disclosure requirements, which require parties and/or candidates to report on political donations, are typically in place where prohibitions on corporate funding are absent or not comprehensive (n_Parties_ = 56). However, these can represent a poor resource for industry monitoring if parties permit anonymous donations (e.g. Sweden), set monetary thresholds on disclosure (n_Parties_ = 29), or do not make reports public (n_Parties_ = 8).

### Reducing the political impacts of corporate social responsibility (Recommendations 6.2-6.4, Article 13)

*6.2 Parties should not endorse support form partnerships with or participate in activities of the tobacco industry described as socially responsible.*

*6.3 Parties should not allow public disclosure by the tobacco industry or any other person acting on its behalf of activities described as socially responsible or of the expenditures made for these activities except when legally required to report on such expenditures such as in an annual report.*

*6.4 Parties should not allow acceptance by any branch of government or the public sector of political social Financial educational community or other contributions from the tobacco industry or from those working to further its interests except for compensations due to legal settlements or mandated by law or legally binding and enforceable agreements.*

*Article 13 Each Party shall, in accordance with its constitution or constitutional principles, undertake a comprehensive ban of all tobacco advertising, promotion and sponsorship.*

In addition to urging Parties to raise awareness of the underlying political purpose of tobacco industry CSR programmes (see above), and providing recommendations regarding partnerships and voluntary initiatives (see above), the guidelines contain a number of further recommendations that explicitly aim to reduce the political impacts of such programmes. Recommendations 6.2 and 6.4 aim to manage industry-government engagement over CSR by urging Parties not to support or endorse industry CSR activities (6.2) and preventing industry actors from providing CSR related contributions to the public sector (6.4). Recommendation 6.3 aims to reduce the agenda setting effect of industry CSR programmes by preventing public disclosure of industry CSR activities and expenditures. In addition, Article 13 requires Parties to ban all forms of sponsorship which, by virtue of sub-section 1(g) of the Article, includes financial and in-kind contributions to community, health, welfare and environmental organizations.

Policy instruments at the national level that explicitly target industry CSR programmes are relatively rare and vary in their coverage. Nepal [111] has a series of overlapping measures in place which collectively appear to ban industry CSR programmes by prohibiting manufacturers and “related parties” from offering “financial, material and structural assistance” and prohibiting industry assistance and collaboration offered in the name of educational development, ethnic or social class advancement and support for emergency services. Ecuador [128] and Thailand [129] have also legislated to ban CSR publicity (6.3) and prohibit government organizations from accepting industry donations (6.4) and, by implication, observe recommendation 6.2. Brazil has measures in place that prohibit members of CONICQ from accepting funding from the tobacco sector and participating in events sponsored by the industry [130]. Suriname [131] and Bangladesh [132] prohibit the use of company and product names, symbols, and trademarks in the context of industry CSR programmes. Public sector organizations in the Philippines are required to disseminate information about the underlying purpose and scope of industry CSR initiatives [73]. Serbia’s Ministry of Health has a policy of not accepting donations and sponsorship from the industry [119]. The Russian Federation prohibits the use of trade names, trademarks and service marks as well as commercial designations belonging to tobacco organizations in the organization and implementation of charitable activities [133].

In practice, formal restraints on industry CSR activities take effect through restrictions on industry sponsorship, awards, donations, and scholarships (hereafter sponsorship unless otherwise stated) in line with Article 13. The degree to which these efforts overlap with the guidelines and restrict industry CSR varies considerably. Some Parties (e.g. Bahrain [134], Djibouti [71], Lao [135], Togo [136]) have prohibited sponsorships outright in respect of all activities, irrespective of whether company names or product trademarks or names are associated with the sponsorship. This approach implies a ban on publicity and largely eliminates the industry’s capacity to use CSR politically. However, it is more common for Parties to impose weaker or more equivocal restrictions on sponsorship, which may permit continued use of CSR by the industry as a means of policy influence, albeit under limited circumstances (Additional file 1: Table S10).

Some Parties impose *de facto* publicity bans on product and corporate sponsorship by prohibiting the use of product trademarks and names, company names, trademarks, symbols, and logos (Additional file 1: Table S10). Prohibition of the use of these restricts effective publicity for CSR, and prevents tobacco companies from using CSR as means of enhancing their reputation. However, it may not affect non-publicised forms of CSR, which can facilitate access and build higher trust relationships with policy actors, enhance companies’ status as a source of credible information with policymakers, and, potentially, build and strengthen constituencies supportive of their position on health policy [24, 27–29, 44, 45, 87]. Indeed, some Parties explicitly make exceptions for such CSR initiatives. Section 11 of the Cook Islands’ Tobacco Act (2007), for example, allows industry actors to support events and organizations, provided association with tobacco companies, products, or brands is “limited to private correspondence” [126]. Further, other Parties (Additional file 1: Table S10) only prohibit the use of product trademarks and names or limit restrictions on sponsorships to circumstances where their purpose is to market or otherwise induce the use of tobacco products, which may permit tobacco manufacturers greater latitude in using CSR politically by permitting corporate publicity (unless otherwise specified) (Additional file 1: Table S10). In some circumstances this is made explicit. South Africa’s Tobacco Products Control Act (1993), for example, preserves the lawfulness of charitable financial contributions and sponsorship, “provided that such contribution or sponsorship is not for the purpose of advertisement.” [137].

Other countries restrict CSR activities to certain issues, such as hunger eradication, poverty reduction, humanitarian activities, social welfare, and the prevention and control of natural disasters [138] but explicitly forbid the industry from publicising these activities in the mass media (Additional file 1: Table S10). By contrast, other Parties restrict prohibition of sponsorship to certain activities such as entertainment, sport, recreational, educational, commercial and cultural purposes and events and activities aimed at minors, leaving open sponsorship for other social issues, such as poverty reduction and health related initiatives (Additional file 1: Table S10).

## Discussion

Despite most Parties reporting some action to implement Article 5.3, our findings indicate that, in practice, implementation is weak and creates extensive opportunities for continued industry policy influence. These opportunities primarily arise from three common features of implementation. First, Parties take a highly selective approach to implementing specific guideline recommendations: only 6% of Parties that have taken some action under Article 5.3 have implemented over half the recommendations. Second, Parties rely heavily on pre-existing policy instruments (passive implementation) governing conflicts of interest in public administration, political financing and lobbying restrictions. These commonly fail to cover all policy actors, frequently omit senior policy actors, and are characterised by exemptions, qualifications, and provisos: all of which create extensive space for continued industry influence. Third, measures introduced in direct response to the FCTC frequently fail to extend beyond health departments, or include third parties in prohibitions and restrictions on industry political activity, or take into account tobacco companies’ capacity to shift the substantive focus of their political activity (from, for instance, youth smoking prevention to the illicit trade in tobacco products).

The policy risks associated with these features are exacerbated by several other characteristics of implementation. These include Parties’ systematic neglect of recommendations that facilitate industry monitoring, which increases the extent to which agent-based opportunities can be used without effective scrutiny. Equally, in many cases implementation demonstrates a lack of joined up thinking, which can further diminish the limited effects of selective action. There is, for instance, less merit in awareness raising measures if parties do not have substantive measures in place to exclude industry actors from health policy-making. Likewise, measures which nominally apply to third parties, are likely to have less effect where actors are not required to disclose their links to tobacco manufacturers. Finally, a number of Parties implement guideline recommendations through working norms. These lack clarity and bureaucratic authority, are easier to challenge than codified working practices, and may leave implementation susceptible to changes in political administration [139].

Relatively widespread misreporting in Party reports suggests that many public officials have a weak understanding of Article 5.3. The fact that some Parties recognise the risks associated with weak implementation also suggests that implementing Article 5.3 may be seen as a low priority by some states [41, 135, 140]. More importantly, Parties’ patchy approach to implementation is premised on a fundamental misunderstanding of how tobacco companies seek to influence policy. The fact that partial implementation limits the universe of possibilities for tobacco companies does not necessarily imply reduced influence. In 2000, for example, industry actors’ ability to access the Prime Minister’s Office in the UK, appeared to weaken health policy even when denied access to other departments [29]. Party reports also suggest that tobacco companies simply lobby more senior policy actors, where restrictions apply to junior officials [135]. Moreover, the number of residual opportunities for policy influence deepens policy risks. Recent research illustrates how tobacco companies in the UK seek to exploit all available opportunities to influence health policy [141]. This heterogeneous quality of political action [18] suggests that gaps in implementation have a cumulative and mutually reinforcing effect on the industry’s ability to build consensus within government and legislatures against policy change. The apparent continuing susceptibility of elected representatives to industry influence is a case in point. [142] In the context of weak implementation of conflict-of-interest provisions [143–147], this permits tobacco companies to build relationships with elected representatives through hospitality and other means [148], which reinforces their efforts to apply political pressure through constituencies in the tobacco supply chain [142]. Finally, the fact that opportunities for policy influence are interdependent, and have the potential to create additional channels of influence, suggests that the type of opportunities left open by partial implementation is also important. These additional channels can expedite relatively subtle changes of emphasis in industry political activity. Partnerships between government and the industry on tobacco tax policy and the illicit trade in tobacco products, for example, can facilitate a range of venue, function, and agent based opportunities for policy influence by facilitating reliance on industry data, closer co-operation between industry actors and government officials, and increased access to policy actors [50, 149–154]. More to the point, they can also facilitate paradigmatic changes in political action. Access to officials involved in brokering trade policy, for example, can shape trade and investment agreements which, in addition to creating new and potentially powerful venues for policy influence by expanding tobacco companies’ access to investor-state dispute settlement procedures [36, 51], can also enable tactical shifts in how the industry lobbies [22, 155].

This last example highlights opportunities for policy influence facilitated by gaps in the guidelines (which do not explicitly address trade and investment agreements) which arise from venue creation. Another important gap concerns Parties’ increasing use of mandatory stakeholder consultations and regulatory impact assessments. Tobacco companies played a key role in embedding both in EU policymaking [54] and continue to lobby trade officials to include them in trade and investment agreements [51]. Stakeholder consultation creates a horizontal, venue-based opportunity for policy influence which circumvents restrictions on government-industry interactions and facilitates agent and functional-based opportunities by opening up the policy process to third parties funded by tobacco companies who draw on reports funded by tobacco companies in their submissions [90, 91, 156]. Finally, the recommendations do not directly cover outsider political strategies [34, 157, 158]. Transparency provisions in the guidelines may help to monitor such strategies, but these are overlooked by the majority of Parties.

## Conclusions

The findings underline the importance of Parties taking an active and whole-of-government approach to Article 5.3 implementation [21], including measures that explicitly cover third parties. There is also a clear case for revisiting the guidelines. Industry political activity is mutable and capable of adapting to altered politico-institutional conditions. Emerging evidence indicates that the guidelines need to take account of innovations in political activity that centre on Better Regulation practices [54, 90–92], and trade and investment agreements [48, 51]. Introducing general duties (in conjunction with specific measures) may partly address this phenomenon, particularly where they apply to policy actors across government [71]. Effective implementation of Article 5.3 also requires Parties to codify the full range of guideline recommendations in administrative measures or legislation. Specific consideration should be given to obligations on tobacco industry actors, in accordance with recommendations 5.2 and 5.3, to submit information concerning their political activities. This would enhance effective industry monitoring, and allow health officials and CSOs to accurately track the industry’s response to restrictions on political activity. Parties may also consider introducing legislative/regulatory footprints, which mandate disclosure of contacts by public and elected officials with stakeholders, and any supporting materials provided by lobbyists [109]. Ideally, this should be a live document, which would help CSOs scrutinise policy-making in real time [109].

There are several pathways to achieving these changes. Parties may consider establishing inter-ministerial bodies aimed at facilitating a whole-of-government approach to implementing the guidelines. Participants must have sufficient institutional capital to push through consequential reforms. [123, 159] CSOs and scholars should also inform public officials of the policy risks attendant on isolated efforts to prohibit or manage specific forms of political activity. Evaluated in terms of its present implementation by Parties, Article 5.3 is largely an expression of symbolic politics [160, 161]. However, as one of several similar documents adopted at sessions of the Conference of the Parties (COP), the governing body of the FCTC, there is agreement that the guidelines constitute a subsequent agreement under Article 31 of the Vienna Convention on the Law of Treaties 1969 [162] and, therefore, should be taken into account by Parties in interpreting their obligations under Article 5.3. The preamble to the guidelines, which states that Parties are “encouraged” to implement them and that their aim is to “assist Parties in meeting their legal obligations” under the Convention, emphasises the non-binding status of the guidelines. Nonetheless, the FCTC’s structure (a primary agreement outlining general principles particularised by detailed guidelines for implementation) and other comments in the guidelines, which encourage Parties to implement measures beyond those outlined, arguably indicates that effective implementation of Article 5.3 not only requires Parties to adopt the guideline recommendations in full, but that this represents the minimum necessary step to giving effect to the Article [24]. Given this, Article 5.3 represents a powerful vehicle around which CSOs and public health professionals could coalesce to advocate material changes in policy. Our findings suggest that this may only occur when the importance of Article 5.3 implementation is more widely recognised and systematically integrated into general efforts aimed at building capacity for successful FCTC implementation [39, 163]. To this end, training on Article 5.3 and its implementation, which takes account of the findings of the present study, is essential [164], as is a deeper understanding that Article 5.3 implementation is an active political process, which necessitates the long term commitment of resources by the WHO, Parties and CSOs.

This last observation is highly relevant to the work of CSOs advocating similar measures be applied to the United Nations Framework Convention on Climate Change [165–168]. The findings presented here are also of relevance to other areas of public health such as alcohol and food, despite the absence of a framework convention in these areas. Article 5.3 and its guidelines are a consequence of sustained analysis and advocacy concerning the socially suboptimal effects of corporate political activity. Translating the findings of this work to detailed guidelines represents an important first step in creating an international political consensus around the value of restricting corporate influence in policy-making and the measures that can be taken to reduce it. However, the findings of this study highlight that this represents a necessary, but not sufficient condition, of effective policy change, that effective implementation and enforcement requires sustained pressure from advocates, health professionals, and lawyers (as well as strong public support), and that internationally agreed frameworks for implementation need to be understood as open and flexible documents which are responsive to innovations in corporate political activity.

Finally, our findings underline the importance of further research exploring the effects of strong implementation of Article 5.3 on general FCTC implementation; the political, cultural and institutional barriers to Article 5.3 implementation; the role of CSOs and other policy entrepreneurs in facilitating Article 5.3 implementation; and the effect of tobacco industry political innovation on health policy. In addition to the kinds of methods used in this article, such research might make effective use of in-depth country-level case studies and key-informant interviews, in order to better understand the processes by which implementation does, or does not, take place [169].

## Additional file

Additional file 1: Table S1. Guidelines for Implementation of Article 5.3 of the FCTC (abridged). **Table S2.** Implementation by Recommendation (all Parties in sample). **Table S3.** Implementation by Recommendation (Parties that have taken some steps in accordance with Article 5.3). **Table S4.** Awareness Raising (Recommendations 1.1, 1.2 and 6.1). **Table S5.** Restricting Access to Policymakers (Recommendation 2.1). **Table S6.** Protocols for Industry-Government Interactions (Recommendation 2.1). **Table S7.** Transparency in Industry Government Interactions (Recommendation 2.2). **Table S8.** Partnerships and Strategic Self-regulations (Recommendations 3.1-3.3). **Table S9.** Policy Subsidies (Recommendations 3.4). **Table S10.** Prohibition of Sponsorship. (DOCX 586 kb)

## Abbreviations

CSO: Civil society organization
CSR: Corporate social responsibility
FCTC: Framework Convention on Tobacco Control
FOI: Freedom of Information
WHO: World Health Organization
