# Supplementary material for: Controlling corporate influence in health policy making? An assessment of the implementation of article 5.3 of the World Health Organization framework convention on tobacco control
Source: Global Health. 2017 Mar 8;13:12. doi: 10.1186/s12992-017-0234-8 (PMC5343400; doi:10.1186/s12992-017-0234-8)
Supplement: Additional file 1: Table S1. — Guidelines for Implementation of Article 5.3 of the FCTC (abridged). Table S2. Implementation by Recommendation (all Parties in sample). Table S3. Implementation by Recommendation (Parties that have taken some steps in accordance with Article 5.3). Table S4. Awareness Raising (Recommendations 1.1, 1.2 and 6.1). Table S5. Restricting Access to Policymakers (Recommendation 2.1). Table S6. Protocols for Industry-Government Interactions (Recommendation 2.1). Table S7. Transparency in Industry Government Interactions (Recommendation 2.2). Table S8. Partnerships and Strategic Self-regulations (Recommendations 3.1-3.3). Table S9. Policy Subsidies (Recommendations 3.4). Table S10. Prohibition of Sponsorship. (DOCX 586 kb) [file 12992_2017_234_MOESM1_ESM.docx]

**Table S1 Guidelines for Implementation of Article 5.3 of the FCTC (abridged)**

| General Recommendations | Specific Recommendations |
| --- | --- |
| Raise awareness about the addictive and harmful nature of tobacco products and about tobacco industry interference with Parties’ tobacco control policies. | 1.1 Parties should inform and educate all branches of government and the public about the addictive and harmful nature of tobacco products, the need to protect public health policies for tobacco control from commercial and other vested interests of the tobacco industry and the strategies and tactics used by the tobacco industry to interfere with the setting and implementation of public health policies with respect to tobacco control. |
|  | 1.2 Parties should, in addition, raise awareness about the tobacco industry’s practice of using individuals, front groups and affiliated organizations to act, openly or covertly, on their behalf or to take action to further the interests of the tobacco industry. |
| Establish measures to limit interactions with the tobacco industry and ensure the transparency of those interactions that occur. | 2.1 Parties should interact with the tobacco industry only when and to the extent strictly necessary to enable them to effectively regulate the tobacco industry and tobacco products. |
|  | 2.2 Where interactions with the tobacco industry are necessary, Parties should ensure that such interactions are conducted transparently. Whenever possible, interactions should be conducted in public, for example through public hearings, public notice of interactions, and disclosure of records of such interactions to the public. |
| Reject partnerships and non-binding or non-enforceable agreements with the tobacco industry. | 3.1 Parties should not accept, support or endorse partnerships and non-binding or non-enforceable agreements as well as any voluntary arrangement with the tobacco industry or any entity or person working to further its interests. |
|  | 3.2 Parties should not accept, support or endorse the tobacco industry organizing, promoting, participating in, or performing, youth, public education or any initiatives that are directly or indirectly related to tobacco control. |
|  | 3.3 Parties should not accept, support or endorse any voluntary code of conduct or instrument crafted by the tobacco industry that is offered as a substitute for legally enforceable tobacco control measures. |
|  | 3.4 Parties should not accept, support or endorse any offer for assistance or proposed tobacco control legislation or policy drafted by or in collaboration with the tobacco industry. |
| Avoid conflicts of interest for government officials and employees. | 4.3 Parties should not award contracts for carrying out any work related to setting and implementing public health policies with respect to tobacco control to candidates or tenderers who have conflicts of interest with established tobacco control policies. |
|  | 4.4 Parties should develop clear policies that require public office holders who have or have had a role in setting and implementing public health policies with respect to tobacco control to inform their institutions about any intention to engage in an occupational activity within the tobacco industry, whether gainful or not, within a specified period of time after leaving service. |
|  | 4.5 Parties should develop clear policies that require applicants for public office positions which have a role in setting and implementing public health policies with respect to tobacco control to declare any current or previous occupational activity with any tobacco industry whether gainful or not. |
|  | 4.6 Parties should require government officials to declare and divest themselves of direct interests in the tobacco industry. |
|  | 4.7 Government institutions and their bodies should not have any financial interest in the tobacco industry, unless they are responsible for managing a Party’s ownership interest in a State-owned tobacco industry. |
|  | 4.8 Parties should not allow any person employed by the tobacco industry or any entity working to further its interests to be a member of any government body, committee or advisory group that sets or implements tobacco control or public health policy. |
|  | 4.9 Parties should not nominate any person employed by the tobacco industry or any entity working to further its interests to serve on delegations to meetings of the Conference of the Parties, its subsidiary bodies or any other bodies established pursuant to decisions of the Conference of the Parties. |
|  | 4.10 Parties should not allow any official or employee of government or of any semi/quasi-governmental body to accept payments, gifts or services, monetary or in-kind, from the tobacco industry. |
|  | 4.11 Taking into account national law and constitutional principles, Parties should have effective measures to prohibit contributions from the tobacco industry or any entity working to further its interests to political parties, candidates or campaigns, or to require full disclosure of such contributions |
| Require that information provided by the tobacco industry be transparent and accurate. | 5.2 Parties should require the tobacco industry and those working to further its interests to periodically submit information on tobacco production, manufacture, market share, marketing expenditures, revenues and any other activity, including lobbying, philanthropy, political contributions and all other activities not prohibited or not yet prohibited under Article 13 of the Convention. |
|  | 5.3 Parties should require rules for the disclosure or registration of the tobacco industry entities, affiliated organizations and individuals acting on their behalf, including lobbyists. |
|  | 5.4 Parties should impose mandatory penalties on the tobacco industry in case of the provision of false or misleading information in accordance with national law. |
| Denormalize and, to the extent possible, regulate activities described as “socially responsible” by the tobacco industry, including but not limited to activities described as “corporate social responsibility”. | 6.1 Parties should ensure that all branches of government and the public are informed and made aware of the true purpose and scope of activities described as socially responsible performed by the tobacco industry. |
|  | 6.2 Parties should not endorse, support, form partnerships with or participate in activities of the tobacco industry described as socially responsible. |
|  | 6.3 Parties should not allow public disclosure by the tobacco industry or any other person acting on its behalf of activities described as socially responsible or of the expenditures made for these activities, except when legally required to report on such expenditures, such as in an annual report. |
|  | 6.4 Parties should not allow acceptance by any branch of government or the public sector of political, social, financial, educational , community or other contributions from the tobacco industry or from those working to further its interests, except for compensations due to legal settlements or mandated by law or legally binding and enforceable agreements. |
| Do not give preferential treatment to the tobacco industry. | 7.2 Parties that do not have a State-owned tobacco industry should not invest in the tobacco industry and related ventures. Parties with a State-owned tobacco industry should ensure that any investment in the tobacco industry does not prevent them from fully implementing the WHO Framework Convention on Tobacco Control. |

# Table S2: Implementation by Recommendation (all parties in sample)

| Recommendation | 1.1 |  | 1.2 |  | 2.1 |  | 2.2 |  | 3.1 |  | 3.2 |  | 3.3 |  | 3.4 |  | 4.3 |  | 4.4 |  | 4.5 |  | 4.6 |  |  |  |
| --- | --- | --- | --- | --- | --- | --- | --- | --- | --- | --- | --- | --- | --- | --- | --- | --- | --- | --- | --- | --- | --- | --- | --- | --- | --- | --- |
|  | n | % | n | % | n | % | n | % | n | % | n | % | n | % | n | % | n | % | n | % | n | % | n | % |  |  |
| Africa | 9 | 24% | 0 | 0% | 1 | 3% | 4 | 11% | 2 | 5% | 2 | 5% | 2 | 5% | 8 | 21% | 0 | 0% | 3 | 8% | 4 | 11% | 20 | 53% |  |  |
| Americas | 10 | 37% | 4 | 15% | 9 | 33% | 7 | 26% | 4 | 15% | 3 | 11% | 3 | 11% | 8 | 30% | 0 | 0% | 3 | 11% | 2 | 7% | 13 | 48% |  |  |
| Asia | 17 | 35% | 4 | 8% | 7 | 14% | 8 | 16% | 12 | 24% | 11 | 22% | 11 | 22% | 11 | 22% | 2 | 4% | 7 | 14% | 3 | 6% | 25 | 51% |  |  |
| Europe | 3 | 7% | 0 | 0% | 7 | 16% | 8 | 19% | 5 | 12% | 6 | 14% | 6 | 14% | 3 | 7% | 1 | 2% | 22 | 51% | 1 | 2% | 27 | 63% |  |  |
| Total | **39** | **25%** | **8** | **5%** | **24** | **15%** | **27** | **17%** | **23** | **15%** | **22** | **14%** | **22** | **14%** | **30** | **19%** | **3** | **2%** | **35** | **22%** | **10** | **6%** | **87** | **55%** |  |  |
| Recommendation | **4.7** |  | **4.8** |  | **4.9** |  | **4.10** |  | **4.11** |  | **5.2** |  | **5.3** |  | **6.1** |  | **6.2** |  | **6.3** |  | **6.4** |  | **7.2** |  | **All** |  |
|  | n | % | n | % | n | % | n | % | n | % | n | % | n | % | n | % | n | % | n | % | n | % | n | % | n | % |
| Africa | 0 | 0% | 18 | 47% | 2 | 5% | 11 | 29% | 9 | 24% | 0 | 0% | 1 | 3% | 0 | 0% | 0 | 0% | 5 | 13% | 1 | 3% | 0 | 0% | 102 | 11% |
| Americas | 0 | 0% | 7 | 26% | 4 | 15% | 5 | 19% | 16 | 59% | 2 | 7% | 4 | 15% | 2 | 7% | 1 | 4% | 2 | 7% | 1 | 4% | 0 | 0% | 107 | 17% |
| Asia | 2 | 4% | 25 | 51% | 6 | 12% | 19 | 39% | 17 | 35% | 1 | 2% | 2 | 4% | 4 | 8% | 5 | 10% | 12 | 24% | 9 | 18% | 3 | 6% | 219 | 19% |
| Europe | 0 | 0% | 27 | 63% | 3 | 7% | 23 | 53% | 22 | 51% | 1 | 2% | 4 | 9% | 0 | 0% | 0 | 0% | 7 | 16% | 1 | 2% | 1 | 2% | 178 | 17% |
| Total | **2** | **1%** | **77** | **49%** | **15** | **10%** | **58** | **37%** | **64** | **41%** | **4** | **3%** | **11** | **7%** | **6** | **4%** | **6** | **4%** | **26** | **17%** | **12** | **8%** | **4** | **3%** | **606** | **16%** |

# Table S3: Implementation by Recommendation (parties that have taken some steps in accordance with Article 5.3)

| Recommendation | 1.1 |  | 1.2 |  | 2.1 |  | 2.2 |  | 3.1 |  | 3.2 |  | 3.3 |  | 3.4 |  | 4.3 |  | 4.4 |  | 4.5 |  | 4.6 |  |  |  |
| --- | --- | --- | --- | --- | --- | --- | --- | --- | --- | --- | --- | --- | --- | --- | --- | --- | --- | --- | --- | --- | --- | --- | --- | --- | --- | --- |
|  | **n** | **%** | **n** | **%** | **n** | **%** | **n** | **%** | **n** | **%** | **n** | **%** | **n** | **%** | **n** | **%** | **n** | **%** | **n** | **%** | **n** | **%** | **n** | **%** |  |  |
| Africa | 9 | 32% | 0 | 0% | 1 | 4% | 4 | 14% | 2 | 7% | 2 | 7% | 2 | 7% | 8 | 29% | 0 | 0% | 3 | 11% | 4 | 14% | 20 | 71% |  |  |
| Americas | 10 | 48% | 4 | 19% | 9 | 43% | 7 | 33% | 4 | 19% | 3 | 14% | 3 | 14% | 8 | 38% | 0 | 0% | 3 | 14% | 2 | 10% | 13 | 62% |  |  |
| Asia | 17 | 43% | 4 | 10% | 7 | 18% | 8 | 20% | 11 | 28% | 11 | 28% | 11 | 28% | 11 | 28% | 2 | 5% | 7 | 18% | 3 | 8% | 25 | 63% |  |  |
| Europe | 3 | 8% | 0 | 0% | 7 | 18% | 8 | 21% | 6 | 16% | 3 | 8% | 6 | 16% | 3 | 8% | 1 | 3% | 22 | 58% | 1 | 3% | 29 | 76% |  |  |
| Total | **39** | **30%** | **8** | **6%** | **24** | **19%** | **27** | **21%** | **23** | **18%** | **30** | **23%** | **22** | **17%** | **30** | **23%** | **3** | **2%** | **35** | **27%** | **10** | **8%** | **87** | **69%** |  |  |
| Recommendation | **4.7** |  | **4.8** |  | **4.9** |  | **4.10** |  | **4.11** |  | **5.2** |  | **5.3** |  | **6.1** |  | **6.2** |  | **6.3** |  | **6.4** |  | **7.2** |  | **All** |  |
|  | **n** | **%** | **n** | **%** | **n** | **%** | **n** | **%** | **n** | **%** | **n** | **%** | **n** | **%** | **n** | **%** | **n** | **%** | **n** | **%** | **n** | **%** | **n** | **%** | **n** | **%** |
| Africa | 0 | 0% | 18 | 64% | 2 | 7% | 11 | 39% | 9 | 32% | 0 | 0% | 1 | 4% | 0 | 0% | 0 | 0% | 5 | 18% | 0 | 0% | 0 | 0% | 102 | 15% |
| Americas | 0 | 0% | 7 | 33% | 4 | 19% | 5 | 24% | 16 | 76% | 2 | 10% | 4 | 19% | 2 | 10% | 1 | 5% | 2 | 10% | 1 | 5% | 0 | 0% | 107 | 21% |
| Asia | 2 | 5% | 25 | 63% | 6 | 15% | 19 | 48% | 17 | 43% | 1 | 3% | 2 | 5% | 4 | 10% | 5 | 10% | 12 | 30% | 7 | 18% | 3 | 8% | 219 | 23% |
| Europe | 0 | 0% | 27 | 71% | 3 | 8% | 23 | 61% | 22 | 58% | 1 | 3% | 4 | 11% | 0 | 0% | 0 | 0% | 7 | 18% | 0 | 0% | 1 | 3% | 178 | 20% |
| Total | **2** | **2%** | **77** | **60%** | **15** | **12%** | **58** | **45%** | **64** | **50%** | **4** | **3%** | **11** | **9%** | **6** | **5%** | **6** | **4%** | **26** | **20%** | **8** | **6%** | **4** | **3%** | **606** | **20%** |

# Table S4: Awareness Raising (Recommendations 1.1, 1.2 and 6.1)*

| Party | Method of Implementation | Audience | Content of awareness raising |
| --- | --- | --- | --- |
| Bahrain | Unspecified | Officials (central)[[1](#_ENREF_1)] | Unspecified |
| Benin | Meeting[[2](#_ENREF_2)] and training (legal advisers)[[3](#_ENREF_3)] | Officials (central and legal advisers), non-governmental actors (CSOs)[[2](#_ENREF_2), [3](#_ENREF_3)] | Unspecified |
| Brazil | Intra-governmental advocacy,[[4](#_ENREF_4)] public awareness raising,[[4](#_ENREF_4)] information sharing[[5](#_ENREF_5)] | Officials (central)[[4](#_ENREF_4)] | All members of CONICQ are required to share information in their possession concerning tobacco industry political activity. Includes awareness raising on CSR and third parties.[[5](#_ENREF_5)] |
| Brunei | Meeting[[6](#_ENREF_6)] | Officials (central), general public[[6](#_ENREF_6)] | Unspecified, but includes awareness raising on third parties.[[6](#_ENREF_6)] |
| Canada | Meeting[[7](#_ENREF_7), [8](#_ENREF_8)] | Officials (central and local/regional)[[7](#_ENREF_7)] | Unspecified |
| Cook Islands | Unspecified | Official (central), elected representatives,[[9](#_ENREF_9)] | Unspecified |
| Costa Rica | Meeting[[10](#_ENREF_10)] | Unspecified | Includes the policy risks attendant on engaging with the industry over illicit trade.[[10](#_ENREF_10)] |
| Cote d’Ivoire | Training (legal advisers)[[11](#_ENREF_11)] | Officials (central and legal advisers), non-governmental actors (CSOs, journalists, and community leaders)[[11](#_ENREF_11)] | Unspecified |
| Ecuador | Meeting and circular[[12](#_ENREF_12)] | Officials (central)[[12](#_ENREF_12)] | Unspecified |
| Estonia | Unspecified | Officials (central)[[13](#_ENREF_13)] | Unspecified |
| Finland | Public awareness raising[[14](#_ENREF_14)] | General public[[14](#_ENREF_14)] | Unspecified |
| Gabon | Meeting[[15](#_ENREF_15)] | Non-governmental actors (CSOs and journalists)[[15](#_ENREF_15)] | Unspecified |
| Ghana | Meeting[[16](#_ENREF_16)] | Officials (central and government agencies and customs officials),[[16](#_ENREF_16)] non-governmental actors (journalists)[[15](#_ENREF_15)] | Unspecified |
| Honduras | Unspecified | Officials (central)[[17](#_ENREF_17)] | Not fully specified, but includes third parties.[[17](#_ENREF_17)] |
| Jamaica | Unspecified | Officials (central)[[18](#_ENREF_18)] | Not fully specified, but includes third parties.[[18](#_ENREF_18)] |
| Kenya | Unspecified | Officials (central)[[19](#_ENREF_19)] | Unspecified |
| Lao | Meeting[[20](#_ENREF_20)] | Officials (central)[[20](#_ENREF_20)] | Unspecified |
| Lebanon | Meeting[[21](#_ENREF_21)] | Officials (central)[[21](#_ENREF_21)] | Unspecified |
| Madagascar | Meeting[[22](#_ENREF_22)] | Officials (central) and non-governmental actors (CSOs and journalists)[[22](#_ENREF_22)] | Unspecified |
| Maldives | Meeting[[23](#_ENREF_23)] | Officials (central)[[23](#_ENREF_23)] | Unspecified |
| Mexico | Unspecified | Officials (central)[[24](#_ENREF_24)] | Unspecified |
| Mongolia | Circular[[25](#_ENREF_25)] and public awareness raising[[25](#_ENREF_25)] | Officials (central)[[25](#_ENREF_25)] the general public[[25](#_ENREF_25)] | Unspecified |
| Myanmar | Meeting[[26](#_ENREF_26)] | Officials (central), non-governmental actors (CSOs)[[26](#_ENREF_26)] | Unspecified |
| Nepal | Unspecified | Unspecified | Not fully specified, but includes CSR awareness raising. |
| Pakistan | Circular[[27](#_ENREF_27)] | Officials (central)[[27](#_ENREF_27)] | Unspecified |
| Palau | Public awareness raising[[28](#_ENREF_28)] | General public[[28](#_ENREF_28)] | Unspecified |
| Panama | Intra-governmental advocacy[[29](#_ENREF_29)] | Officials (central)[[30](#_ENREF_30)] | General compliance with Article 5.3 and its guidelines, includes CSR awareness raising.[[29](#_ENREF_29)] |
| Philippines | Public awareness raising [[31](#_ENREF_31)] | Officials (central and local/regional), the general public [[31-33](#_ENREF_31)] | Not fully specified, but includes third parties CSR awareness raising.[[31](#_ENREF_31)] |
| Solomon Islands | Training based measures (civil servants)[[34](#_ENREF_34)] | Officials (central)[[34](#_ENREF_34)] | Unspecified |
| South Africa | Unspecified | Officials (central)[[35](#_ENREF_35)] | Unspecified |
| Spain | Unspecified | Officials (central)[[36](#_ENREF_36)] | Unspecified |
| Suriname | Unspecified | Officials (central)[[37](#_ENREF_37)] | Unspecified |
| Thailand | Public awareness raising[[38](#_ENREF_38), [39](#_ENREF_39)] | General public[[38](#_ENREF_38), [39](#_ENREF_39)] | Not fully specified, but includes third parties and CSR awareness raising[[39](#_ENREF_39)] |
| United Kingdom | Circular[[40](#_ENREF_40)] | Officials (central and local/regional)[[40](#_ENREF_40)] | Unspecified |
| Vietnam | Circular, meetings, intra-governmental advocacy[[41](#_ENREF_41)] | Officials (central)[[41](#_ENREF_41)] | Unspecified, but includes third parties and CSR[[41](#_ENREF_41)] |

*Only includes parties where data includes information on the method of awareness raising, its audience, or its content.

# Table S5: Restricting Access to Policymakers (Recommendation 2.1)*

| Party | Method of Implementation | Institutional Reach | Range of Actors Covered |
| --- | --- | --- | --- |
| Antigua and Barbuda | Working norm[[42](#_ENREF_42)] | Ministry of Health or department/agency within ministry[[42](#_ENREF_42)] | Tobacco industry[[42](#_ENREF_42)] |
| Australia | Legal instrument/code of practice[[43](#_ENREF_43)] | Ministry of Health or department/agency within ministry[[43](#_ENREF_43)] | Tobacco industry[[43](#_ENREF_43)] |
| Bahrain | Legal instrument/code of practice[[1](#_ENREF_1)] | Unspecified[[1](#_ENREF_1)] | Tobacco industry[[1](#_ENREF_1)] |
| Bulgaria | Working norm[[44](#_ENREF_44)] | Ministry of Health or department/agency within ministry[[44](#_ENREF_44)] | Tobacco industry[[44](#_ENREF_44)] |
| Canada | Working norm[[8](#_ENREF_8)] | Ministry of Health or department/agency within ministry[[8](#_ENREF_8)] | Tobacco industry[[8](#_ENREF_8)] |
| Ecuador | Working norm[[12](#_ENREF_12)] | Ministry of Health or department/agency within ministry[[12](#_ENREF_12)] | Tobacco industry[[12](#_ENREF_12)] |
| Finland | Working norm[[14](#_ENREF_14)] | Ministry of Health or department/agency within ministry[[14](#_ENREF_14)] | Tobacco industry[[14](#_ENREF_14)] |
| Gabon | Legal instrument/code of practice[[45](#_ENREF_45)] | Across government[[45](#_ENREF_45)] | Tobacco industry[[45](#_ENREF_45)] |
| Honduras | Legal instrument/code of practice[[17](#_ENREF_17)] | Ministry of Health or department/agency within ministry[[17](#_ENREF_17)] | Tobacco industry[[17](#_ENREF_17)] |
| Ireland | Working norm[[46](#_ENREF_46)] | Ministry of Health or department/agency within ministry[[46](#_ENREF_46)] | Tobacco industry[[46](#_ENREF_46)] |
| Mexico | Legal instrument/code of practice[[24](#_ENREF_24)] | Unspecified[[24](#_ENREF_24)] | Tobacco industry[[24](#_ENREF_24)] |
| Nepal | Working norm[[47](#_ENREF_47)] | Ministry of Health or department/agency within ministry[[47](#_ENREF_47)] | Tobacco industry and third parties[[47](#_ENREF_47)] |
| Netherlands | Working norm[[48](#_ENREF_48)] | Across government[[48](#_ENREF_48)] | Tobacco industry[[48](#_ENREF_48)] |
| Pakistan | Legal instrument/code of practice[[49](#_ENREF_49)] | Unspecified[[49](#_ENREF_49)] | Tobacco industry[[49](#_ENREF_49)] |
| Panama | Legal instrument/code of practice[[29](#_ENREF_29)] | Committee for the Study of Tobacco within the Directorate General of Public Health[[29](#_ENREF_29)] | Tobacco industry[[29](#_ENREF_29)] |
| Paraguay | Working norm[[50](#_ENREF_50)] | Ministry of Health or department/agency within ministry[[50](#_ENREF_50)] | Tobacco industry[[50](#_ENREF_50)] |
| Philippines | Legal instrument/code of practice[[31](#_ENREF_31), [32](#_ENREF_32)] | Across government[[31](#_ENREF_31), [32](#_ENREF_32)] | Tobacco industry and third parties[[31](#_ENREF_31), [32](#_ENREF_32)] |
| St Lucia | Working norm[[51](#_ENREF_51)] | Unspecified[[51](#_ENREF_51)] | Tobacco industry[[51](#_ENREF_51)] |
| Singapore | Legal instrument/code of practice[[52](#_ENREF_52)] | Across government[[52](#_ENREF_52)] | Tobacco Industry[[52](#_ENREF_52)] |
| Spain | Legal instrument/code of practice[[36](#_ENREF_36)] | Unspecified[[36](#_ENREF_36)] | Tobacco industry[[36](#_ENREF_36)] |
| Thailand | Legal instrument/code of practice[[39](#_ENREF_39), [53](#_ENREF_53)] | Ministry of Health or department/agency within ministry[[39](#_ENREF_39), [53](#_ENREF_53)] | Tobacco industry and third parties[139] |
| Uruguay | Working norm[[54](#_ENREF_54)] | Ministry of Health or department/agency within ministry[[54](#_ENREF_54)] | Tobacco industry[[54](#_ENREF_54)] |
| UK | Legal instrument/code of practice[[40](#_ENREF_40)] | Across government[[40](#_ENREF_40)] | Tobacco industry[[40](#_ENREF_40)] |

*Only includes parties where data included information on the method of implementation, institutional reach or range of actors covered.

# Table S6: Protocols for Industry-Government Interactions (Recommendation 2.1)

| Party | Rules on Interaction | Institutional Reach | Range of Actors Covered |
| --- | --- | --- | --- |
| Australia | Requirement of a lawyer present, at least two departmental officers present, meetings minuted.[[55](#_ENREF_55)] | Ministry of Health or department/agency within ministry[[55](#_ENREF_55)] | Tobacco industry[[55](#_ENREF_55)] |
| Brazil | Requirement of written notice, restrictions on participation to industry actors named in advanced in writing, at least two departmental officers present, meetings take place on government premises, meetings minuted.[[5](#_ENREF_5)] | National commission for FCTC implementation (CONIQ)[[5](#_ENREF_5)] | Tobacco industry[[5](#_ENREF_5)] |
| Nepal | At least two departmental officers present[[56](#_ENREF_56)] | Ministry of Health or department/agency within ministry[[56](#_ENREF_56)] | Tobacco industry[[56](#_ENREF_56)] |
| Pakistan | Meetings minuted[[57](#_ENREF_57)] | Ministry of Health or department/agency within ministry[[57](#_ENREF_57)] | Tobacco industry[[57](#_ENREF_57)] |
| Panama | Ministry of Health (pre-determined agenda, meetings minuted[[29](#_ENREF_29)]); across government (meetings minuted and attended an official from the Ministry of Health.[[30](#_ENREF_30)] | Directorate within health ministry[[29](#_ENREF_29)] and across government[[30](#_ENREF_30)] | Ministry of health (tobacco industry and third parties[[29](#_ENREF_29)]); across government (unspecified) |
| Philippines | Requirement of written notice, restriction of meetings to specific issues, pre-determined agenda, restrictions on participation to industry actors named in advanced in writing, voice recordings, requirement of a lawyer present, meetings take place on government premises.[[31](#_ENREF_31)] | Ministry of Health/agency within ministry[[31](#_ENREF_31)] | Tobacco industry and third parties[[31](#_ENREF_31)] |
| Singapore | Pre-determined agenda, meetings minuted[[58](#_ENREF_58)] | Ministry of Health or department/agency within ministry[[58](#_ENREF_58)] | Tobacco Industry[[58](#_ENREF_58)] |
| Solomon Islands | Meetings minuted[[59](#_ENREF_59)] | Across government[[59](#_ENREF_59)] | Tobacco Industry and third parties[[59](#_ENREF_59)] |
| Thailand | Requirement of written notice, restriction of meetings to specific issues, pre-determined agenda, meetings take place on government premises, meetings minuted.[[53](#_ENREF_53)] | Health ministry[[53](#_ENREF_53)] | Tobacco industry and third parties[[53](#_ENREF_53)] |

# Table S7: Transparency in Industry Government Interactions (Recommendation 2.2)*

| Parties | Method of Implementation | Institutional Reach | Range of Actors Covered |
| --- | --- | --- | --- |
| Australia | Publication of details and minutes of meetings and submissions to public consultations[[60](#_ENREF_60)] | Ministry of Health or department/agency within ministry,[[60](#_ENREF_60)] Australian Taxation Office[[61](#_ENREF_61)] | Tobacco industry[[60](#_ENREF_60)] |
| Bahrain | Unspecified[[1](#_ENREF_1)] | Across government departments[[1](#_ENREF_1)] | Tobacco industry[[1](#_ENREF_1)] |
| Brazil | Unspecified[[5](#_ENREF_5)] | National Commission for  the Implementation of the Framework Convention on Tobacco Control and its  Protocols (CONIQ)[[5](#_ENREF_5)] | Tobacco industry and third parties[[5](#_ENREF_5)] |
| Burkina Faso | Unspecified[[62](#_ENREF_62)] | Across government departments[[62](#_ENREF_62)] | Tobacco industry[[62](#_ENREF_62)] |
| Canada | Publication of details of meetings[[7](#_ENREF_7)] | Across government departments[[7](#_ENREF_7)] | Tobacco industry and third parties[[7](#_ENREF_7)] |
| Colombia | Third party direct observation[[63](#_ENREF_63)] | Ministry of Health or department/agency within ministry[[63](#_ENREF_63)] | Tobacco industry[[63](#_ENREF_63)] |
| Gabon | Unspecified[[64](#_ENREF_64)] | Across government departments[[64](#_ENREF_64)] | Tobacco industry and third parties[[64](#_ENREF_64)] |
| Ghana | Publication of details of meetings[[65](#_ENREF_65)] | Across government departments[[65](#_ENREF_65)] | Tobacco industry and third parties[[65](#_ENREF_65)] |
| India | Publication of details of meeting on request[[66](#_ENREF_66)] | Across government departments[[66](#_ENREF_66)] | Tobacco industry and third parties[[66](#_ENREF_66)] |
| Kenya | Requirement for health officials to be notified[[67](#_ENREF_67)] | Across government departments[[67](#_ENREF_67)] | Tobacco industry[[67](#_ENREF_67)] |
| Latvia | Draft laws that come before the Latvian parliament must enclose an explanatory note which specifies all consultations held while preparing the bill.[[68](#_ENREF_68)] | Across government departments[[68](#_ENREF_68)] | Tobacco industry and third parties[[68](#_ENREF_68)] |
| Mexico | Third party direct observation and publication of details of meetings on request[[69](#_ENREF_69)] | Across government departments[[70](#_ENREF_70)] | Tobacco industry[[70](#_ENREF_70)] |
| Netherlands | Publication of details of meetings on request[[48](#_ENREF_48)] | Across government department [[48](#_ENREF_48)] | Tobacco industry[[48](#_ENREF_48)] |
| Norway | Publication of communications relating to consultations concerning proposed health policies[[71](#_ENREF_71)] | Unspecified[[71](#_ENREF_71)] | Tobacco industry and third parties[[71](#_ENREF_71)] |
| Nepal | Third party direct observation[[56](#_ENREF_56)] | Across government departments[[56](#_ENREF_56)] | Tobacco industry[[56](#_ENREF_56)] |
| New Zealand | Publication of details of meetings and publication of submissions to public consultations[[72](#_ENREF_72), [73](#_ENREF_73)] | Ministry of Health or department/agency within ministry[[72](#_ENREF_72), [73](#_ENREF_73)] | Tobacco industry[[72](#_ENREF_72), [73](#_ENREF_73)] |
| Panama | Minutes[[29](#_ENREF_29)] | National Committee for the Study of Tobacco Use within the Directorate General of Public Health[[29](#_ENREF_29)] | Tobacco industry[[29](#_ENREF_29)] |
| Philippines | Transcripts of meetings and correspondence[[31](#_ENREF_31), [32](#_ENREF_32)] | Across government departments[[32](#_ENREF_32)] | Tobacco industry and third parties[[32](#_ENREF_32)] |
| Poland | Publication of all documents related to drafting of legislation.[[68](#_ENREF_68)] | Across government departments[[68](#_ENREF_68)] | Tobacco industry third parties[[68](#_ENREF_68)] |
| Russian Federation | Publication of written government responses to electronic correspondence by tobacco companies[[74](#_ENREF_74)] | Across government departments[[74](#_ENREF_74)] | Unspecified |
| Slovakia | Publication of lobbying contact reports (excluding the name of the lobbyist)[[68](#_ENREF_68)] | Across government departments[[68](#_ENREF_68)] | Tobacco industry and third parties[[68](#_ENREF_68)] |
| Spain | Unspecified[[36](#_ENREF_36)] | Unspecified[[36](#_ENREF_36)] | Unspecified[[36](#_ENREF_36)] |
| Thailand | Publication of details of meetings[[53](#_ENREF_53)] | Ministry of Health or department/agency within ministry[[39](#_ENREF_39), [53](#_ENREF_53)] | Tobacco industry and third parties[[53](#_ENREF_53)] |
| Turkey | Unspecified[[75](#_ENREF_75)] | Unspecified[[75](#_ENREF_75)] | Unspecified[[75](#_ENREF_75)] |
| United Kingdom | Transcripts of meetings and correspondence made publicly available. Publication of submissions to public consultations.[[76](#_ENREF_76)] | Across government departments and agencies (with some exceptions)[[76](#_ENREF_76)] | Tobacco industry and third parties[[76](#_ENREF_76)] |
| Uruguay | Third party direct observation[[54](#_ENREF_54)] | Unspecified[[54](#_ENREF_54)] | Tobacco Industry[[54](#_ENREF_54)] |

*Only includes parties where data included information on the method of implementation, institutional reach or range of actors covered.

# Table S8: Partnerships and Strategic Self-regulations (Recommendations 3.1-3.3)*

| Parties | Partnerships or non-binding or non-enforceable Agreements (3.1) | Youth, public education or other initiatives related to tobacco control (3.2) | Voluntary Codes (3.3) |
| --- | --- | --- | --- |
| Bahrain** | Working norm[[1](#_ENREF_1)] | None reported in additional questions[[1](#_ENREF_1)] | Working norm[[1](#_ENREF_1)] |
| Belize** | Working norm[[77](#_ENREF_77)] | None reported in additional questions[[77](#_ENREF_77)] | Working norm[[77](#_ENREF_77)] |
| Brazil | Administrative rule (applicable to pan-governmental tobacco control body)[[5](#_ENREF_5)] | Administrative rule (applicable to pan-governmental tobacco control body)[[5](#_ENREF_5)] | Administrative rule (applicable to pan-governmental tobacco control body)[[5](#_ENREF_5)] |
| Brunei** | Working norm[[6](#_ENREF_6)] | None reported in additional questions[[6](#_ENREF_6)] | Working norm[[6](#_ENREF_6)] |
| Canada | Administrative rule[[7](#_ENREF_7)] | Administrative rule[[7](#_ENREF_7)] | Administrative rule[[7](#_ENREF_7)] |
| Colombia** | Working norm[[63](#_ENREF_63)] | No evidence of endorsement[[63](#_ENREF_63)] | Working norm[[63](#_ENREF_63)] |
| Costa Rica** | Working norm[[78](#_ENREF_78)] | None reported in additional questions[[78](#_ENREF_78)] | Working norm[[78](#_ENREF_78)] |
| Gabon** | Primary Legislation[[64](#_ENREF_64)] | Primary Legislation[[64](#_ENREF_64)] | Primary Legislation[[64](#_ENREF_64)] |
| Ghana** | Working norm[[16](#_ENREF_16)] | None reported in additional questions[[16](#_ENREF_16)] | Working norm [[16](#_ENREF_16)] |
| Honduras** | Working norm[[17](#_ENREF_17)] | Primary Legislation[[17](#_ENREF_17)] | Working norm[[17](#_ENREF_17)] |
| Jamaica** | Voluntary arrangement with the tobacco industry prohibiting advertising tobacco products  in print media targeting children.[[79](#_ENREF_79)] | No evidence of endorsement[[79](#_ENREF_79)] | No evidence of endorsement[[79](#_ENREF_79)] |
| Japan** | Working norm[[80](#_ENREF_80)] | None reported in additional questions[[80](#_ENREF_80)] | Working norm[[80](#_ENREF_80)] |
| Latvia** | Non-Compliance[[81](#_ENREF_81)] | No evidence of endorsement[[81](#_ENREF_81)] | No evidence of endorsement[[81](#_ENREF_81)] |
| Mexico** | Working norm[[24](#_ENREF_24)] | None reported in additional questions[[24](#_ENREF_24)] | Working norm[[24](#_ENREF_24)] |
| Mongolia | Primary Legislation[[82](#_ENREF_82)] | Primary Legislation[[82](#_ENREF_82)] | Primary Legislation[[82](#_ENREF_82)] |
| Montenegro | No evidence | De facto rejection[[83](#_ENREF_83)] | No evidence |
| Nepal | Regulatory Directive[[56](#_ENREF_56)] | Regulatory Directive[[56](#_ENREF_56)] | Regulatory Directive[[56](#_ENREF_56)] |
| Norway** | Working norm[[71](#_ENREF_71), [84](#_ENREF_84)] | None reported in additional questions[[71](#_ENREF_71), [84](#_ENREF_84)] | Working norm[[71](#_ENREF_71), [84](#_ENREF_84)] |
| Pakistan** | Working norm[[49](#_ENREF_49)] | None reported in additional questions[[49](#_ENREF_49)] | Working norm[[49](#_ENREF_49)] |
| Panama** | Evidence unclear[[30](#_ENREF_30)] | None reported in additional questions[[30](#_ENREF_30)] | Working norm[[30](#_ENREF_30)] |
| Philippines | Cross departmental code of conduct and department of health memorandum[[31](#_ENREF_31)] | Cross departmental code of conduct and department of health memorandum[[31](#_ENREF_31)] | Cross departmental code of conduct and department of health memorandum[[31](#_ENREF_31)] |
| Serbia | Code of Practice[[85](#_ENREF_85)] | Code of Practice[[85](#_ENREF_85)] | No evidence[[85](#_ENREF_85)] |
| Spain** | Working norm[[36](#_ENREF_36)] | None reported in additional questions[[36](#_ENREF_36)] | Working norm[[36](#_ENREF_36)] |
| Thailand | Administrative rule[[39](#_ENREF_39)] | Administrative rule[[39](#_ENREF_39)] | Administrative rule[[39](#_ENREF_39)] |
| Togo | No evidence | Primary Legislation[[64](#_ENREF_64)] | No evidence |
| Tonga** | Working norm[[86](#_ENREF_86)] | None reported in additional questions[[86](#_ENREF_86)] | Working norm[[86](#_ENREF_86)] |
| Turkey** | Working norm[[75](#_ENREF_75)] | None reported additional questions[[75](#_ENREF_75)] | Working norm[[75](#_ENREF_75)] |
| Ukraine** | Working norm[[87](#_ENREF_87)] | Primary Legislation (banned)[[87](#_ENREF_87)] | Working norm[[87](#_ENREF_87)] |

*Only includes parties with codified measures and who have provided responses to the additional questions.

**Parties submitting answers to additional questions on the use of implementation guidelines adopted by the Conference of the Parties.[[88](#_ENREF_88)]

# Table S9: Policy Subsidies (Recommendations 3.4)*

| Party | Method of Implementation | Institutional Reach | Range of Actors Covered |
| --- | --- | --- | --- |
| Antigua and Barbuda | *Ad hoc* action[[42](#_ENREF_42), [89](#_ENREF_89)] | Ministry of Health or department/agency within ministry[[42](#_ENREF_42), [89](#_ENREF_89)] | Tobacco industry[[42](#_ENREF_42), [89](#_ENREF_89)] |
| Bahrain | Working norm[[1](#_ENREF_1), [90](#_ENREF_90)] | Ministry of Health or department/agency within ministry[[1](#_ENREF_1), [90](#_ENREF_90)] | Tobacco industry[[1](#_ENREF_1), [90](#_ENREF_90)] |
| Belize | No offer reported in additional questions[[77](#_ENREF_77)] | Ministry of Health or department/agency within ministry[[77](#_ENREF_77)] | Tobacco industry[[77](#_ENREF_77)] |
| Brazil | Administrative rule[[5](#_ENREF_5)] | National commission for FCTC implementation (CONIQ)[[5](#_ENREF_5)] | Tobacco industry[[5](#_ENREF_5)] |
| Brunei | No offer reported in additional questions[[6](#_ENREF_6)] | Ministry of Health or department/agency within ministry[[6](#_ENREF_6)] | Tobacco industry[[6](#_ENREF_6)] |
| Canada | Administrative rule[[8](#_ENREF_8)] | Ministry of Health or department/agency within ministry[[8](#_ENREF_8)] | Tobacco industry[[8](#_ENREF_8)] |
| China | Working norm[[91](#_ENREF_91)] | Ministry of Health or department/agency within ministry[[91](#_ENREF_91)] | Tobacco industry[[91](#_ENREF_91)] |
| Colombia | *Ad hoc* action[[92](#_ENREF_92)] | Ministry of Health or department/agency within ministry[[92](#_ENREF_92)] | Tobacco industry[[92](#_ENREF_92)] |
| Costa Rica | No offer reported in additional questions[[78](#_ENREF_78)] | Ministry of Health or department/agency within ministry[[78](#_ENREF_78)] | Tobacco industry[[78](#_ENREF_78)] |
| Djibouti | Primary Legislation[[93](#_ENREF_93)] | Cross departmental[[93](#_ENREF_93)] | Tobacco industry[[93](#_ENREF_93)] |
| Ecuador | Working norm[[12](#_ENREF_12)] | Ministry of Health or department/agency within ministry[[12](#_ENREF_12)] | Tobacco industry[[12](#_ENREF_12)] |
| Gabon | Primary Legislation[[64](#_ENREF_64)] | Ministry of Health or department/agency within ministry[[64](#_ENREF_64)] | Tobacco industry and third parties[[64](#_ENREF_64)] |
| Ghana | Working norm[[65](#_ENREF_65)] | Ministry of Health or department/agency within ministry[[65](#_ENREF_65)] | Tobacco industry[[65](#_ENREF_65)] |
| Honduras | Primary Legislation[[17](#_ENREF_17), [94](#_ENREF_94)] | Ministry of Health or department/agency within ministry[[17](#_ENREF_17), [94](#_ENREF_94)] | Tobacco industry[[17](#_ENREF_17), [94](#_ENREF_94)] |
| India | Administrative rule[[95](#_ENREF_95)] | Ministry of Health or department/agency within ministry[[95](#_ENREF_95)] | Tobacco industry[[95](#_ENREF_95)] |
| Jamaica | Working norm[[18](#_ENREF_18), [79](#_ENREF_79)] | Ministry of Health or department/agency within ministry[[18](#_ENREF_18), [79](#_ENREF_79)] | Tobacco industry[[18](#_ENREF_18), [79](#_ENREF_79)] |
| Japan | No offer reported in additional questions[[80](#_ENREF_80)] | Ministry of Health or department/agency within ministry[[80](#_ENREF_80)] | Tobacco industry[[80](#_ENREF_80)] |
| Lebanon | *Ad hoc* rejection[[21](#_ENREF_21)] | Ministry of Health or department/agency within ministry[[21](#_ENREF_21)] | Tobacco industry[[21](#_ENREF_21)] |
| Madagascar | Working norm[[22](#_ENREF_22)] | Ministry of Health or department/agency within ministry[[22](#_ENREF_22)] | Tobacco industry[[22](#_ENREF_22)] |
| Mali | *Ad hoc* rejection[[96](#_ENREF_96)] | Ministry of Health or department/agency within ministry[[96](#_ENREF_96)] | Tobacco industry[[96](#_ENREF_96)] |
| Mauritius | *Ad hoc* rejection[[97](#_ENREF_97)] | Ministry of Health or department/agency within ministry[[97](#_ENREF_97)] | Tobacco industry[[97](#_ENREF_97)] |
| Mexico | No offer reported in additional questions[[24](#_ENREF_24), [30](#_ENREF_30)] | Ministry of Health or department/agency within ministry[[24](#_ENREF_24), [30](#_ENREF_30)] | Tobacco industry[[24](#_ENREF_24), [30](#_ENREF_30)] |
| Mongolia | Primary legislation[[82](#_ENREF_82)] | Ministry of Health or department/agency within ministry[[82](#_ENREF_82)] | Tobacco industry and third parties[[82](#_ENREF_82)] |
| Nepal | Secondary legislation[[56](#_ENREF_56)] | Ministries of Health, Finance, Revenue Collection, and Trade[[48](#_ENREF_48)] | Tobacco industry and third parties[[48](#_ENREF_48)] |
| Netherlands | Working norm[[48](#_ENREF_48)] | Ministry of Health or department/agency within ministry[[48](#_ENREF_48)] | Tobacco industry[[48](#_ENREF_48)] |
| Pakistan | Working norm[[49](#_ENREF_49), [57](#_ENREF_57)] | Ministry of Health or department/agency within ministry[[49](#_ENREF_49), [57](#_ENREF_57)] | Tobacco industry[[49](#_ENREF_49), [57](#_ENREF_57)] |
| Panama | Primary Legislation[[30](#_ENREF_30), [94](#_ENREF_94)] | Ministry of Health or department/agency within ministry[[30](#_ENREF_30), [94](#_ENREF_94)] | Tobacco industry[[30](#_ENREF_30), [94](#_ENREF_94)] |
| Peru | *Ad hoc* action[[94](#_ENREF_94)] | Ministry of Health or department/agency within ministry[[94](#_ENREF_94)] | Tobacco industry[[94](#_ENREF_94)] |
| Senegal | Primary legislation[[98](#_ENREF_98)] | Ministry of Health or department/agency within ministry[[98](#_ENREF_98)] | Tobacco industry[[98](#_ENREF_98)] |
| Sierra Leone | Working norm[[99](#_ENREF_99)] | Ministry of Health or department/agency within ministry[[99](#_ENREF_99)] | Tobacco industry[[99](#_ENREF_99)] |
| St Lucia | Working norm[[51](#_ENREF_51)] | Ministry of Health or department/agency within ministry[[51](#_ENREF_51)] | Tobacco industry[[51](#_ENREF_51)] |
| Spain | No offer reported in additional questions[[36](#_ENREF_36)] | Ministry of Health or department/agency within ministry[[36](#_ENREF_36)] | Tobacco industry[[36](#_ENREF_36)] |
| Swaziland | Working norm[[100](#_ENREF_100)] | Ministry of Health or department/agency within ministry[[100](#_ENREF_100)] | Tobacco industry and third parties[[100](#_ENREF_100)] |
| Thailand | Administrative rule[[39](#_ENREF_39)] | Ministry of Health or department/agency within ministry[[39](#_ENREF_39)] | Tobacco industry[[39](#_ENREF_39)] |
| Turkey | Primary Legislation[[75](#_ENREF_75)] | Ministry of Health or department/agency within ministry[[75](#_ENREF_75)] | Tobacco industry[[75](#_ENREF_75)] |

*Only includes parties where data included information on the method of implementation, institutional reach or range of actors covered.

# Table S10: Prohibition of Sponsorship*

| Party | Limited to marketing, promoting or inducing the use of tobacco products | Specification of product names and trademarks, | Specification of company names/trademarks | Exemptions on Activities: entertainment, sport, recreation, education, culture |
| --- | --- | --- | --- | --- |
| Bahrain | No[[1](#_ENREF_1)] | Not specified | Not specified | None |
| Bangladesh** | Yes[[101](#_ENREF_101)] | Yes | Yes | None |
| Belarus | No[[102](#_ENREF_102)] | Yes | Yes | None |
| Belgium | Yes[[103](#_ENREF_103)] | Not specified | Not specified | None |
| Bosnia | Yes[[104](#_ENREF_104)] | Not specified | Not specified | None |
| Brazil** | Yes[[105](#_ENREF_105)] | Not specified | Not specified | Restricted to cultural and sporting activities.[[105](#_ENREF_105)] |
| Brunei | Yes[[106](#_ENREF_106)] | Not specified | Not specified | None |
| Burkina Faso | Yes[[62](#_ENREF_62)] | Not specified | Not specified | None |
| Cambodia | Yes[[107](#_ENREF_107)] | Yes | Not specified | Permitted for humanitarian activities or activities for social benefits in which tobacco commercial interests or tobacco advertising is not shown.[[107](#_ENREF_107)] |
| Chad | Yes[[108](#_ENREF_108)] | Not specified | Not specified | None |
| Colombia | Yes[[109](#_ENREF_109)] | Not specified | Not specified | Restricted to sporting and cultural events.[[109](#_ENREF_109)] |
| Cook Islands | No[[110](#_ENREF_110)] | Yes | Yes | None |
| Costa Rica | Yes[[111](#_ENREF_111)] | Not specified | Not specified | None |
| Czech Republic | Yes[[112](#_ENREF_112)] | Not specified | Not specified | None |
| Denmark | Yes[[113](#_ENREF_113)] | Not specified | Not specified | None |
| Djibouti | No[[93](#_ENREF_93)] | Not specified | Not specified | None |
| Ecuador** | Yes[[114](#_ENREF_114), [115](#_ENREF_115)] | Not specified | Not specified | None |
| Estonia | Yes[[116](#_ENREF_116)] | Not specified | Not specified | None |
| Finland | Yes[[117](#_ENREF_117)] | Yes | Not specified | None |
| France | Yes[[118](#_ENREF_118)] | Not specified | Not specified | None |
| Gabon | Yes[[64](#_ENREF_64)] | Yes | No | Restricted to sporting, artistic or other “events”.[[116](#_ENREF_116)] |
| Ghana | No[[119](#_ENREF_119)] | Yes | Yes | None |
| Honduras | Yes[[120](#_ENREF_120)] | Not specified | Not specified | None |
| Hungary | No[[121](#_ENREF_121)] | Not specified | Not specified | Prohibited in connection with sporting and cultural events, and events or activities relating to health care or organised by political parties.[[121](#_ENREF_121)] |
| Iceland | Yes[[122](#_ENREF_122)] | Not specified | Not specified | Restricted to events and activities.[[122](#_ENREF_122)] |
| India | Yes[[123](#_ENREF_123)] | Yes | Not specified | None |
| Iran | Yes[[124](#_ENREF_124)] | Not specified | Not specified | None |
| Ireland | Yes[[125](#_ENREF_125)] | Not specified | Not specified | None |
| Kenya | Yes[[126](#_ENREF_126)] | Not specified | Not specified | None |
| Kuwait | No[[127](#_ENREF_127)] | Not specified | Not specified | None |
| Lao | No[[20](#_ENREF_20)] | Not specified | Not specified | None |
| Lebanon | Yes[[128](#_ENREF_128)] | Not specified | Not specified | Restricted to cultural, sporting and commercial events.[[128](#_ENREF_128)] |
| Lithuania | Yes[[129](#_ENREF_129)] | Not specified | Not specified | None |
| Macedonia | No[[130](#_ENREF_130)] | Yes | Not specified | Restricted to sports, cultural, entertainment and other public performances and events.[[130](#_ENREF_130)] |
| Madagascar | Yes[[131](#_ENREF_131)] | Not specified | Not specified | None |
| Maldives | No[[132](#_ENREF_132)] | Not specified | Not specified | None |
| Mali | Yes[[133](#_ENREF_133)] | Not specified | Not specified | None |
| Mauritius | No[[134](#_ENREF_134)] | Yes | Yes | None |
| Mexico | Yes[[135](#_ENREF_135)] | Yes | Not specified | None |
| Moldova | Yes[[136](#_ENREF_136)] | Yes | Yes | None |
| Mongolia | No[[82](#_ENREF_82)] | Yes | Yes | None |
| Montenegro | Yes[[137](#_ENREF_137)] | Not specified | Not specified | None |
| Namibia | No[[138](#_ENREF_138)] | Yes | Yes | Restricted to entertainment, sport, recreation, education, culture.[[56](#_ENREF_56), [138](#_ENREF_138)] |
| Nepal** | Yes[[56](#_ENREF_56)] | Not specified | Not specified | None |
| New Zealand | No[[139](#_ENREF_139)] | Yes | Yes | Prohibition of use of product and company trademarks restricted services, activities, events, scholarships, fellowships or education.[[139](#_ENREF_139)] |
| Niger | Yes[[140](#_ENREF_140)] | Yes | Not specified | None |
| Norway | Yes[[141](#_ENREF_141)] | Yes | No | None |
| Palau | Yes[[142](#_ENREF_142)] | Yes | Yes | None |
| Panama | Yes[[143](#_ENREF_143)] | Not specified | Not specified | None |
| Poland | No[[144](#_ENREF_144)] | Yes | Yes | Prohibition of tobacco sponsorship of “sport, cultural, educational, health, social, and political activities” (but not necessarily groups and/or individuals).[[144](#_ENREF_144)] |
| Portugal | Yes[[145](#_ENREF_145)] | Not specified | Not specified | None |
| Peru | Yes[[146](#_ENREF_146)] | Yes | Not specified | Restricted to events or activities aimed at minors.[[146](#_ENREF_146)] |
| Philippines** | Yes[[147](#_ENREF_147)] | Yes | Yes | Tobacco companies prohibited from sponsoring any sport, concert, cultural or art event, as well as individual and team athletes, artists or performers which involves the advertisement or promotion of a tobacco company, tobacco product or tobacco use, name, logo or trademarks and other words, symbols, designs, colours or other depictions commonly associated with or likely to identify a tobacco product. The attribution only to the name of the company in a roster of sponsors is permitted.[[147](#_ENREF_147)] |
| Romania | Yes[[148](#_ENREF_148)] | Not specified | Not specified | Prohibition restricted to events and activities for young people under the age of 18 years and the sponsorship of tobacco products to any events or activities taking place in health units or units of medical and pharmaceutical education.[[148](#_ENREF_148)] |
| Russia** | Yes[[149](#_ENREF_149)] | Not specified | Not specified | None |
| Senegal | No[[98](#_ENREF_98)] | Yes | Yes | Restricted to sport and culture.[[98](#_ENREF_98)] |
| Serbia** | No | No | No | Tobacco companies prohibited from sponsoring underage persons, their activities, or any persons or activities the audience of which consists mainly of underage persons, media, sports persons, sports contests or other contests or any individual persons or participants in such events.[[150](#_ENREF_150)] |
| Seychelles | Yes[[151](#_ENREF_151)] | Not specified | Not specified | None |
| Singapore | Yes[[152](#_ENREF_152)] | Yes | Yes | None |
| South Africa | Yes[[153](#_ENREF_153)] | Yes | Yes | Permitted for charitable financial contribution or sponsorship, provided that such contribution or sponsorship is not for the purpose of advertisement.[[153](#_ENREF_153)] |
| Spain | Yes[[154](#_ENREF_154)] | Not specified | Not specified | None |
| Suriname** | Yes[[155](#_ENREF_155)] | Not specified | Not specified | None |
| Swaziland | Yes[[156](#_ENREF_156)] | Not specified | Not specified | None |
| Thailand** | Yes[[157](#_ENREF_157)] (although sponsorship not specified) | Not specified | Not specified | None |
| Togo | No[[158](#_ENREF_158)] | Not specified | Not specified | The relevant decree prohibits the provision of financial or other support for events or activities, involving private individuals or groups, whether or not it is in exchange for publicity, particularly philanthropic activities of companies, or programs for the prevention of tobacco use among young people.[[158](#_ENREF_158)] |
| Tonga | No[[159](#_ENREF_159)] | Yes | Yes | None |
| Turkey | No[[160](#_ENREF_160)] | Yes | Yes | Companies may not use their names, logos, emblems, products brands, trademarks, or symbols in publicizing financial or other support provided to an event or an activity. However, there does not appear to be any restrictions on sponsorship to things other than events or activities, and therefore publicity of sponsorship of individuals and organizations may be allowed.[[160](#_ENREF_160)] |
| Tuvalu | No[[161](#_ENREF_161)] | Yes | Yes | None |
| Ukraine | Yes[[162](#_ENREF_162)] | Not specified | Not specified | None |
| United Kingdom | Yes[[163](#_ENREF_163)] | Not specified | Not specified | None |
| Uruguay | Yes[[164](#_ENREF_164)] | Not specified | Not specified | None |
| Vanuatu | No[[165](#_ENREF_165)] | Yes | Yes | Restricted to events and activities.[[165](#_ENREF_165)] |
| Vietnam | No[[166](#_ENREF_166)] | Not specified | Not specified | Permitted for programmes of hunger eradication and poverty reduction;  prevention and control of natural disasters, epidemics, disasters;  and for combating cigarette smuggling.[[166](#_ENREF_166)] |

*Excludes parties where prohibitions on sponsorship are limited to specific media (e.g. digital or radio) or age-limited.

** Denotes parties who have additional provisions in place which specifically relate to CSR (see main text).

# References (Tables)

1. Public Health Directorate Ministry of Health (Bahrain): *Additional Questions on the Use of Implementation Guidelines by the Parties.* Manama: Public Health Directorate, Ministry of Health; 2014.

2. Point Focal Tabac Alcool et Drogues (Benin): *Instrument de Notification de law Convention-Cadre de l'OMS pour la Lutte Antitabac.* Cotonou: Ministere de la Sante; 2014.

3. Alcool Direction Nationale de la Santé Publique Ministère de la Santé (Républic du Bénin): **Email Correspondence.** 2015.

4. Executive Secretariat of the National Commission for the Implementation of the Framework Convention for Tobacco Control: *Reporting Instrument of the WHO Framework Convention on Tobacco Control.* Rio de Janeiro: National Cancer Institute; 2010.

5. **Administrative Rule N^o^713, 2012**.

6. National Focal Point on Tobacco Control (Brunei Darussalam): *Additional Questions on the Use of Implementation Guidelines by the Parties*. Bandar Seri Begawan: Ministry of Health; 2014.

7. Controlled Substances and Tobacco Directorate (Canada): *Reporting Instrument of the WHO Framework Convention on Tobacco Control.* Ottawa: Health Canada; 2014.

8. Controlled Substances and Tobacco Directorate Health Canada (Canada): *Reporting Instrument under the WHO Framework Convention on Tobacco Control.* Ottawa: Health Canada; 2010.

9. Cook Islands Ministry of Health: *Cook Islands Tobacco Control Action Plan 2012-2016.* Avarua: Cook Islands Ministry of Health; 2012.

10. Responsable del Programa Control del Tabaco Ministerio de Salud (Costa Rica): **Email Correspondence.** 2015.

11. Programme National de Lutte contre le Tabagisme l'Alcoolisme la Toxicomanie et les autres Addictions (Côte d'Ivoire): *Instrument de Notification de la Convention-Cadre de l'OMS pour la Lutte Antitabac.* Yamoussoukro: Ministère de la Santé et de la Lutte contre le Sida; 2014.

12. Ministerio de Salud Pública (Ecuador): *Instrumento de Presentaciòn de Informes del Convenio Marco de la OMS para el Control del Tabaco.* Quito: Ministerio de Salud Pública; 2014.

13. Ministry of Social Affairs (Estonia): *Reporting Instrument of the WHO Framework Convention on Tobacco Control.* Tallinn: Ministry of Social Affairs; 2014.

14. Ministry of Social Affairs and Health (Finland): *Reporting Instrument of the WHO Framework Convention on Tobacco Control.* Helsinki: Ministry of Social Affairs and Health; 2014.

15. People's Health Movement Gabon (PHM-Gabon): *Réunion de Restitution de l’atelier 5.3 de Lomé et de .3 de Lomé et de Planification des activités de Lutte antitabac au Gabon: RAPPORT FINAL.* Libreville: Mouvement Populaire pour la Sante au Gabon (MPS-Gabon) 2013.

16. Disease Control and Prevention Department (Ghana): *Additional Questions on the Use of Implementation Guidelines by the Parties.* Accra: Ghana Health Service; 2014.

17. Instituto Hondureňo para la Prevención del Alcoholismo Drogadicciòn y Farmacodependencia (Honduras): *Preguntas Adicionales sobre el uso por las Partes de las Directrices para la Aplicación.* Tegucigalpa: Ministerio de Salud; 2014.

18. Ministry of Health (Jamaica): *Reporting Instrument of the WHO Framework Convention on Tobacco Control.* Kingston: Ministry of Health; 2014.

19. Division of Noncommunicable Diseases (Kenya): *Reporting Instrument of the WHO Framework Convention on Tobacco Control.* Nairobi: Ministry of Health; 2014.

20. Hygiene and Health Promotion Department (Lao People's Democratic Republic): *Reporting Instrument of the WHO Framework Convention on Tobacco Control.* Vientiane: Ministry of Health; 2014.

21. National Tobacco Control Program (Lebanon): *Reporting Instrument of the WHO Framework Convention on Tobacco Control.* Beirut: Ministry of Health; 2014.

22. Office National de Lutte Antitabac (Madagascar): *Instrument de Notification de la Convention-Cadre de l'OMS pour la Lutte Antitabac.* Antananarivo: Ministère de la Santé Publique; 2014.

23. Health Protection Agency (Maldives): *Reporting Instrument on the WHO Framework Convention on Tobacco Control.* Malé: Ministry of Health and Gender 2014.

24. Comisión Nacional Contra las Adicciones Centro Nacional para la Prevención y el Control de las Adicciones (Mexico): *Preguntas Adicionales sobre el uso por las Partes de las Directrices para la Aplicación.* Mexico City: Comisión Nacional Contra las Adicciones Centro Nacional para la Prevención y el Control de las Adicciones; 2014.

25. Public Health Division of the Policy Implementation and Coordination Department (Mongolia): *Reporting Instrument of the WHO Framework Convention on Tobacco Control.* Ulaanbaata: Ministry of Health; 2014.

26. Department of Health, Ministry of Health (Myanmar): *Reporting Instrument of the WHO Framework Convention on Tobacco Control.* Naypyidaw: Ministry of Health; 2014.

27. Tobacco Control Cell, Ministry of National Health Services Regulations and Coordination (Pakistan): **Email Correspondence.** 2015.

28. Tobacco Use Prevention and Control Program (Palau): *Reporting Instrument of the WHO Framework Convention on Tobacco Control.* Koror: Ministry of Health; 2014.

29. **Resolución No.745 (National Commission for the Study of Tobacco), 2012**.

30. Focal de Control de Tabaco (Panama): *Preguntas Adicionales sobre el uso por las Partes de las Directrices para la Aplicación.* Ministerio de Salud; 2014.

31. Department of Health (Philippines): **Department of Health Memorandum No. 2010-0126 on Protection of the Department of Health, including all of its Agencies, Regional Offices, Bureaus or Specialized/Attached Offices/Units, against Tobacco Industry Interference.** Manila: Department of Health; 2010.

32. Civil Service Commission (Philippines), Department of Health (Philippines): **Joint Memorandum Circular No. 2010-01 on Protection of the Bureaucracy Against Tobacco Industry Interference.** ((Philippines) CSC, (Philippines) DoH eds.). Manila: Civil Service Commission and Department of Health; 2010.

33. Department of Health (Philippines): *Reporting Instrument of the WHO Framework Convention on Tobacco Control.* Manila: Department of Health; 2014.

34. NCDs/National Tobacco Control Focal Point (Solomon Islands): *Reporting Instrument of the WHO Framework Convention on Tobacco Control.* Honiara: Ministry of Health and Medical Services; 2014.

35. Alcohol and Drug Abuse Research Unit South African Medical Research Council (South Africa): *Reporting Instrument of the WHO Framework Convention on Tobacco Control.* Pretoria: National Department of Health; 2014.

36. Ministerio de Sanidad Servicios Sociales e Igualdad (Spain): *Preguntas Adicionales sobre el uso por las Partes de las Directrices para la Aplicación.* Madrid: Ministerio de Sanidad, Servicios Sociales e Igualdad 2014.

37. Ministry of Health (Suriname): *Reporting Instrument of the WHO Framework Convention on Tobacco Control.* Paramaribo: Ministry of Health (Suriname); 2014.

38. Ministry of Public Health (Thailand): *Reporting Instrument of the WHO Framework Convention on Tobacco Control.* Thivanoth: Bureau of Tobacco Control Department of Disease Control, Ministry of Public Health; 2010.

39. Ministry of Public Health (Thailand): *Reporting Instrument of the WHO Framework Convention on Tobacco Control.* Thivanoth: Bureau of Tobacco Control Department of Disease Control, Ministry of Public Health; 2014.

40. Tobacco Programme, Department of Health (United Kingdom): *Reporting Instrument of the WHO Framework Convention on Tobacco Control.* London: Department of Health; 2014.

41. Standing Office of Vietnam Steering Committee on Smoking and Health. Ministry of Health (Vietnam): *Reporting Instrument of the WHO Framework Convention on Tobacco Control.* Hanoi: Ministry of Health; 2014.

42. Health Information Division, Ministry of Health (Antigua and Barbuda): *Reporting Instrument of the WHO Framework Convention on Tobacco Control.* St Johns: Ministry of Health; 2014.

43. Department of Health (Australian Government): *Department of Health Internal Policy - Interactions with the Tobacco Industry.* Canberra: Department of Health; undated.

44. Ministry of Health (Bulgaria): *Reporting Instrument of the WHO Framework Convention on Tobacco Control.* Sofia: Ministry of Health; 2012.

45. Programme National de Santé Mentale et de Lutte contre le Tabac l’Alcoolisme et les Drogues Ministère de la Santé (Gabon): *Questions supplémentaires sur l’utilisation des directives d’application par les parties*. Libreville: Ministère de la Santé; 2014.

46. Tobacco and Alcohol Control Unit (Ireland): *Reporting Instrument of the WHO Framework Convention on Tobacco Control.* Dublin: Department of Health; 2014.

47. National Health Education Information and Communication Centre, Ministry of Health (Nepal): *Reporting Instrument of the WHO Framework Convention on Tobacco Control.* Kathmandu: Ministry of Health; 2014.

48. Ministry of Health Welfare and Sports (Netherlands): *Reporting Instrument of the WHO Framework Convention on Tobacco Control.* The Hague: Ministry of Health, Welfare and Sports; 2014.

49. Tobacco Control Cell Ministry of National Health Services (Pakistan): *Additional Questions on the Use of Implementation Guidelines by the Parties.* Islamabad: Ministry of National Health Services; 2014.

50. Ministerio de Salud Pública y Bienestar Social (Paraguay): *Instrumento de Presentaciòn de Informes del Convenio Marco de la Oms para el Control del Tabaco.* Asunción: Ministerio de Salud Pública y Bienestar Social; 2014.

51. Substance Abuse Advisory Council Secretariat, Ministry of Health (Sierra Leone): *Reporting Instrument of the WHO Framework Convention on Tobacco Control.* Castries: Ministry of Health; 2012.

52. Health Promotion Board, Ministry of Health (Singapore): *Reporting Instrument of the WHO Framework Convention on Tobacco Control.* Singapore: Ministry of Health; 2014.

53. **Regulation of Department of Disease Control Regarding How to Contact Tobacco Entrepreneurs and Related Persons B.E. 2553, 2010**.

54. Ministerio de Salud Pública (Uruguay): *Instrumento de Presentaciòn de Informes del Convenio Marco de la Oms para el Control del Tabaco.* Montevideo: Ministerio de Salud Pública; 2014.

55. Department of Health (Australian Government): **Department of Health Internal Policy - Interactions with the Tobacco Industry.** Canberra: Department of Health; undated.

56. **Tobacco Product Control and Regulatory Directive, 2014**.

57. Tobacco Control Cell, Ministry of National Health Services (Pakistan): *Reporting Instrument of the WHO Framework Convention on Tobacco Control.* Islamabad: Ministry of National Health Services; 2014.

58. Health Promotion Board (Singapore): *Reporting Instrument of the WHO Framework Convention on Tobacco Control.* Singapore: Health Promotion Board; 2012.

59. Solomon Islands Government: *Code of Conduct. Solomon Islands Public Service.* Ministry of Public Service; 2009.

60. **Public notification of meetings between the Australian Government Department of Health and Ageing and the Tobacco industry** [<http://www.health.gov.au/internet/main/publishing.nsf/Content/tobacco-conv-public>]. Accessed April 15 2015.

61. **Tobacco Stakeholder Group** [<https://www.ato.gov.au/General/Consultation/Consultation-groups/Stakeholder-relationship-management-groups/Tobacco-Stakeholder-Group/>]. Accessed April 4 2016.

62. **Law No. 040-2010/AN, Concerning the Campaign against Tobacco in Burkina Faso, 2010**.

63. Ministerio de Salud y Proteccion (Colombia): *Preguntas Adicionales sobre el uso por las Partes de las Directrices para la Aplicación.* Carrera; 2014.

64. **Law No. 006/2013 of August 21, 2013, Concerning the Enactment of Measures Supporting the Campaign for Tobacco Control in the Republic of Gabon, 2013**.

65. Department of Health (Ghana): *Reporting Instrument of the WHO Framework Convention on Tobacco Control.* Accra: Department of Health; 2014.

66. **Right to Information Act, 2005**.

67. Ministry of Public Health and Sanitation (Kenya): *Phase 2 (Group 2 Questions) of the Reporting Instrument under the WHO Framework Convention on Tobacco Control.* Nairobi: Ministry of Public Health and Sanitation; 2010.

68. Mulcahy S: *Lobbying in the EU.* Berlin: Transparency International; 2015.

69. Centro Nacional para la Prevención y Control de las Adicciones: *Instrumento de Presentaciòn de Informes del Convenio Marco de la OMS para el Control del Tabaco.* Mexico City: Secretaría de Salud; 2012.

70. da Costa e Silva VL, Bialous SA: *Technical Resource for Country Implementation of WHO Framework Convention on Tobacco Control Article 5.3.* Geneva: World Health Organization; 2012.

71. Norwegian Directorate of Health: *Additional Questions on the Use of Implementation Guidelines by the Parties*. Oslo: Norwegian Directorate of Health; 2014.

72. Tobacco control Programme (New Zealand): *Reporting Instrument of the WHO Framework Convention on Tobacco Control.* Wellington: Ministry of Health; 2014.

73. **Meetings with tobacco industry representatives** [<http://www.health.govt.nz/our-work/preventative-health-wellness/tobacco-control/who-framework-convention-tobacco-control/meetings-tobacco-industry-representatives>]. Accessed April 4 2016.

74. **Federal Law N 15-FZ of February 23, 2013 On Protecting the Health of Citizens from the Effects of Second Hand Tobacco Smoke and the Consequences of Tobacco Consumption, 2013**.

75. Tobacco and Other Addictive Drugs Control Department (Turkey): *Additional Questions on the Use of Implementation Guidelines by the Parties* Ankara: Ministry of Health; 2014.

76. **Publications: FOI Releases** [<https://www.gov.uk/government/publications?keywords=tobacco&publication_filter_option=foi-releases&topics%5B%5D=all&departments%5B%5D=department-of-health&official_document_status=all&world_locations%5B%5D=all&from_date=&to_date=>]. Accessed April 16 2015.

77. National Drug Abuse Control Council (Belize): *Additional Questions on the Use of Implementation Guidelines by the Parties.* Belize City: Ministry of Health; 2014.

78. Ministerio de Salud de Costa Rica: *Preguntas Adicionales sobre el uso por las Partes de las Directrices para la Aplicación.* San José: Ministerio de Salud de Costa Rica; 2014.

79. Ministry of Health (Jamaica): *Additional Questions on the Use of Implementation Guidelines by the Parties* Kingston: Ministry of Health; 2014.

80. Ministry of Health Labour and Welfare (Japan): *Additional Questions on the Use of Implementation Guidelines by the Parties* Tokyo: Ministry of Health, Labour and Welfare; 2014.

81. Ministry of Health (Latvia): *Additional Questions on the Use of Implementation Guidelines by the Parties*. Riga: Ministry of Health; 2014.

82. **Tobacco Control Law, 2012**.

83. Institute of Public Health (Montenegro): *Reporting Instrument of the WHO Framework Convention on Tobacco Control.* Podgorica: Ministry of Health; 2014.

84. Norwegian Ministry of Health and Care Services: *Consultation on the proposal for standardised tobacco packaging and the implementation of Article 5.3 of the Framework Convention on Tobacco Control.* Oslo: Norwegian Ministry of Health and Care Services; 2015.

85. Ministry of Health (Serbia): *Reporting Instrument of the WHO Framework Convention on Tobacco Control.* Belgrade: Ministry of Health; 2014.

86. Public Health Division, Ministry of Health (Tonga): *Additional Questions on the Use of Implementation Guidelines by the Parties*. Nukuʻalofa: Ministry of Health; 2014.

87. Ukrainian Institute for Strategic Research of the Ministry of Health (Ukraine): *Additional Questions on the Use of Implementation Guidelines by the Parties* Kiev: Ministry of Health; 2014.

88. **Reporting Instrument** [<http://www.who.int/fctc/reporting/reporting_instrument/en/>]. Accessed May 14 2015.

89. Health Information Division (Antigua and Barbuda): *Reporting Instrument of the WHO Framework Convention on Tobacco Control.* St Johns: Ministry of Health; 2012.

90. Bahrain Ministry of Health: *Reporting Instrument of the WHO Framework Convention on Tobacco Control.* Manama: Antismoking Group, Bahrain Ministry of Health; 2014.

91. Ministry of Consumer Goods Industry (China): *Reporting Instrument of the WHO Framework Convention on Tobacco Control.* Beijing: Ministry of Consumer Goods Industry; 2014.

92. Corporate Accountability International. **The Global Tobacco Treaty** [<http://www.stopcorporateabuse.org/global-tobacco-treaty>]. Accessed April 13 2015.

93. **Law n°175/AN/07/5ème L concerning organization for the protection of health against the tobacco habit, 2007**.

94. InterAmerican Heart Foundation: *Framework Convention on Tobacco Control: challenges for Latin America and the Caribbean Civil Society Report.* Dallas, TX: InterAmerican Heart Foundation; 2010.

95. Ministry of Health & Family Welfare (India): *Reporting Instrument of the WHO Framework Convention on Tobacco Control.* New Delhi: Ministry of Health and Family Welfare; 2012.

96. Direction Nationale de la Sante (Mali): *Instrument de Notification de la Convetion-Cadre de l'Oms pour la Lutte Antitabac.* Bamako: Direction Nationale de la Santé; 2014.

97. Ministry of Health and Quality of Life (Mauritius): *Phase 2 (Group 2 Questions) of the Reporting Instrument under the WHO Framework Convention on Tobacco Control.* Port Louis,: Ministry of Health and Quality of Life; 2010.

98. **Law concerning the manufacture, packaging, labeling, sale and use of tobacco, 2014.**

99. Health Education Division (Sierra Leone): *Reporting Instrument of the WHO Framework Convention on Tobacco Control.* Freetown: Ministry of Health and Sanitation; 2014.

100. Ministry of Health (Swaziland): *Reporting Instrument of the WHO Framework Convention on Tobacco Control.* Mbabane: Ministry of Health; 2012.

101. **Act No.16 of 2013, An Act for the Amendment of Smoking and Tobacco Products Usage (Control) Act, 2005**.

102. **Law of the Republic of Belarus 10 May 2007, No. 225-З on Advertising, 2007**.

103. **Law prohibiting the advertising of tobacco products, 1997**.

104. **Law on the Ban on Advertising of Tobacco Products, 2004.**

105. **Law No. 9.294, of July 15, 1996.**

106. **Tobacco Order, 2005**.

107. **Sub-decree on Advertising of Tobacco Products, 2014**.

108. **Law No.010/PR/2010 on Tobacco Control, 2010**.

109. **Provisions by which Damages caused to Minors and the Non-Smoking Population are Prevented and Public Policies are Stipulated to Prevent Tobacco Consumption and the Cessation of the Smoker's Dependence on Tobacco and its Derivatives in the Colombian People, 2009**.

110. **Tobacco Products Control Act, 2007**.

111. **General Law for the Control of Tobacco and its Harmful Effects on Health, 2012**.

112. **Act No. 40-1995, On the Regulation of Advertising (as amended).**

113. **Act to Consolidate the Law Prohibiting Advertising etc. about Tobacco Products, 2008**.

114. **Organic Law for the Regulation and Control of Tobacco, 2011**.

115. **Regulation to the Tobacco Regulation and Control Act, No.1047, 2012**.

116. **Tobacco Act, 2005**.

117. **Tobacco Act (No. 693-1976) (as amended through 2010), 1976**.

118. **Code of Public Health, 2013**.

119. **Public Health Act, 2012**.

120. **Special Tobacco Control Law, 2010**.

121. **Act XLVIII of 2008 on the Basic Requirements of and Certain Restrictions on Commercial Advertising Activities, 2008**.

122. **Tobacco Control Act No.6/2002, 2002**.

123. **Cigarettes and Other Tobacco Products (Prohibition of Advertisement and Regulation of Trade and Commerce, Production, Supply and Distribution) Act, 2003**.

124. **Executive Bylaw of Comprehensive Act on National Control and Campaign Against Tobacco**, **2007**.

125. **Public Health (Tobacco) Act, 2002**.

126. **Tobacco Control Act, 2007**.

127. **Examples of implementation of Article 5.3 communicated through the reports of the Parties** [<http://www.who.int/fctc/parties_experiences/en/index.html>]. Accessed April 10 2015.

128. **Law No.174, Tobacco Control and Regulation of Tobacco Products’ Manufacturing, Packaging and Advertising, 2011**.

129. **Law on Tobacco Control, 1995** (amended 2011).

130. **Law on Protection against Smoking (Consolidated version (1995-2010)**.

131. **Interministerial Order No. 18171, Laying Down the Rules on Industrialization, Importation, Marketing and Consumption of Tobacco Products in Madagascar, 2003**.

132. **Tobacco Control Act, 2010**.

133. **Law No. 10-033 of July 12, 2010, On the Sale and Consumption of Tobacco and Tobacco Products, 2010**.

134. **Regulations made by the Minister under sections 193 and 194 of the Public Health Act, 2008**.

135. **General Law on Tobacco Control (Ley General para el Control del Tabaco), 2008**.

136. **Law No. 124 for Amendment and Supplementation of Certain Legislative Acts (including amendments to Law No. 278-XVI on Tobacco and Tobacco Products, Law No. 1227-XIII on advertising, and Criminal Code No. 218-XVI), 2007**.

137. **Law on Amendments to the Law on restriction of the usage of tobacco products, 2011**.

138. **Tobacco Products Control Act, 2010**.

139. **Smoke-free Environments Act, 1990**.

140. **Law No. 2006-12 of May 15 Concerning Tobacco Control, 2006**.

141. **Regulations no. 989 of December 15, 1995, On the Prohibition of advertising of tobacco products etc.**

142. **World Health Organizations Framework Convention on Tobacco Control, RPPL 8-27 2011, 2009.**

143. **Law of No.13 of January 24, 2008 Which Adopts Measures to Control Tobacco and its Harmful Effects on Health.**

144. **Protection of Public Health against the Effects of Tobacco Use, 1995.**

145. **Law No.37/2007, 2007.**

146. **Regulations Approval of Law No 28705, General Law on Prevention and Control of the Risks of Tobacco Use Supreme Decree, 2008.**

147. **Tobacco Regulation Act, 2003**.

148. **Law No. 457 of November 1, 2004, On Advertising and Sponsorship for Tobacco Products (as amended)**.

149. **Federal Law N 15-FZ of February 23, 2013, On Protecting the Health of Citizens from the Effects of Second Hand Tobacco Smoke and the Consequences of Tobacco Consumption**.

150. **Law on Advertising, 2005**.

151. **Tobacco Control Act, 2009**.

152. **Tobacco (Control of Advertisements and Sale) Act, 2011**.

153. **Tobacco Products Control Act, 1993** **(as amended)**.

154. **Law 28/2005, of December 26, 2005, Regarding Health Measures against Tobacco Addiction and Regulating Sales, Supplies, Consumption and Advertising of Tobacco Products**.

155. **Act of February 20, 2013, Laying Down Rules Limiting the Use of Tobacco and Tobacco Products (Tobacco Act), 2013**.

156. **The Tobacco Products Control Act**.

157. **Tobacco Products Control Act, B.E. 2535, 1992**.

158. **Decree No. 2012-072, Concerning the Prohibition of Advertising, Promotion and Sponsorship of Tobacco and its Derivative Products in Togo, 2012**.

159. **Tobacco Control Act, 2000**.

160. **The Law on Prevention and Control of Harzards of Tobacco Products, 1996**.

161. **Tobacco Control Act, 2008**.

162. **On the Introduction of Changes to Some Legislative Acts of Ukraine on the Prohibition of the Advertising, Sponsorship and Promotion of the Sale of Tobacco Products, 2012**.

163. **Tobacco Advertising and Promotion Act, 2002**.

164. **Law No. 18,256 Smoking Control Regulations, 2008**.

165. **Tobacco Control Act No.19 of 2008.**

166. **Law on Prevention and Control of Tobacco Harms, 2012**.
